# Single-cell mapping of alternative splicing linked to checkpoint immunotherapy response

**DOI:** 10.1093/nar/gkaf1171

**Published:** 2025-11-20

**Authors:** Jieyi Xiong, Orian Bricard, Ingrid Arijs, Jonas Demeulemeester, Chen Gu, Bernard Thienpont, Danie Daaboul, Ayse Bassez, Oliver Bechter, Joanna Poźniak, Hanne Vos, Ines Nevelsteen, Sofie Torfs, Sara Aibar Santos, Qing Lan, Yong Hou, Lore Van Oudenhove, Gitta Boons, Junbin Qian, Stein Aerts, Ann Smeets, Jean-Christophe Marine, Diether Lambrechts

**Affiliations:** Laboratory for Translational Genetics, Department of Human Genetics, KU Leuven, 3000 Leuven, Belgium; VIB Center for Cancer Biology, 3000 Leuven, Belgium; Laboratory for Translational Genetics, Department of Human Genetics, KU Leuven, 3000 Leuven, Belgium; VIB Center for Cancer Biology, 3000 Leuven, Belgium; Laboratory for Translational Genetics, Department of Human Genetics, KU Leuven, 3000 Leuven, Belgium; VIB Center for Cancer Biology, 3000 Leuven, Belgium; VIB Center for Cancer Biology, 3000 Leuven, Belgium; Laboratory for Integrative Cancer Genomics, Department of Oncology, KU Leuven, 3000 Leuven, Belgium; VIB Center for AI & Computational Biology (VIB.AI), 3000 Leuven, Belgium; Laboratory for Translational Genetics, Department of Human Genetics, KU Leuven, 3000 Leuven, Belgium; VIB Center for Cancer Biology, 3000 Leuven, Belgium; Laboratory for Functional Epigenetics, Department of Human Genetics, KU Leuven, 3000 Leuven, Belgium; VIB Center for AI & Computational Biology (VIB.AI), 3000 Leuven, Belgium; VIB Center for Brain & Disease Research, 3000 Leuven, Belgium; Laboratory of Computational Biology, Department of Human Genetics, KU Leuven, 3000 Leuven, Belgium; Laboratory for Translational Genetics, Department of Human Genetics, KU Leuven, 3000 Leuven, Belgium; VIB Center for Cancer Biology, 3000 Leuven, Belgium; Department of General Medical Oncology, University Hospitals Leuven, 3000 Leuven, Belgium; VIB Center for Cancer Biology, 3000 Leuven, Belgium; Laboratory for Molecular Cancer Biology, Department of Oncology, KU Leuven, 3000 Leuven, Belgium; Department of Surgical Oncology, University Hospitals Leuven, KU Leuven, 3000 Leuven, Belgium; Department of Surgical Oncology, University Hospitals Leuven, KU Leuven, 3000 Leuven, Belgium; Laboratory for Translational Genetics, Department of Human Genetics, KU Leuven, 3000 Leuven, Belgium; VIB Center for Cancer Biology, 3000 Leuven, Belgium; VIB Center for AI & Computational Biology (VIB.AI), 3000 Leuven, Belgium; VIB Center for Brain & Disease Research, 3000 Leuven, Belgium; Laboratory of Computational Biology, Department of Human Genetics, KU Leuven, 3000 Leuven, Belgium; BGI-Research, Shenzhen, 518085, China; BGI-Research, Shenzhen, 518085, China; myNEO Therapeutics, 9000 Ghent, Belgium; myNEO Therapeutics, 9000 Ghent, Belgium; Zhejiang Key Laboratory of Precision Diagnosis and Therapy for Major Gynecological Diseases, Women’s Hospital, Zhejiang University School of Medicine, Hangzhou, 310029, China; Institute of Genetics, Zhejiang University School of Medicine, Hangzhou, 310058, China; VIB Center for AI & Computational Biology (VIB.AI), 3000 Leuven, Belgium; VIB Center for Brain & Disease Research, 3000 Leuven, Belgium; Laboratory of Computational Biology, Department of Human Genetics, KU Leuven, 3000 Leuven, Belgium; Department of Surgical Oncology, University Hospitals Leuven, KU Leuven, 3000 Leuven, Belgium; VIB Center for Cancer Biology, 3000 Leuven, Belgium; Laboratory for Molecular Cancer Biology, Department of Oncology, KU Leuven, 3000 Leuven, Belgium; Laboratory for Translational Genetics, Department of Human Genetics, KU Leuven, 3000 Leuven, Belgium; VIB Center for Cancer Biology, 3000 Leuven, Belgium

## Abstract

Evidence suggests that alternative RNA splicing (AS) plays a critical role in tumor biology and may contribute to the generation of tumor antigens. Here, we develop a method to detect AS in short-read single-cell 5′-RNA-sequencing data, allowing us to uniquely characterize the heterogeneity and dynamic changes in AS in individual cell types within the tumor microenvironment. We identify numerous splicing events specific to either cancer cells or stromal cell types or for triple-negative versus estrogen receptor-positive breast cancers (BCs). By correlating these splice events with expression of splicing regulators in individual cells, we also identify their potential mediators. For instance, we identify and functionally validate the Epithelial Splicing Regulatory Protein-1 (ESRP1) to drive AS in BCs responding to immune checkpoint blockade (ICB). Prioritization of splicing events based on their likelihood to represent tumor antigens reveals that their aggregated load also correlates with high immune activity in multiple cancers, while also predicting expansion of T cells in BCs receiving ICB and prolonging long-term survival of cancer patients treated with ICB. Collectively, our method provides a framework for analyzing AS in single-cell data and defines a key role for AS in the response to ICB.

## Graphical Abstract

**Graphical Abstract:**
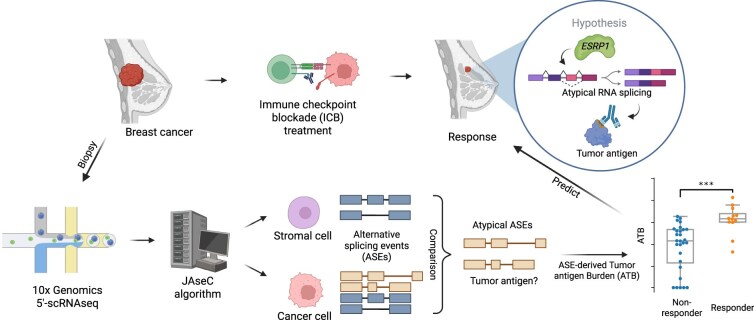

## Introduction

RNA splicing is an evolutionarily conserved process that plays a key role in human health and disease. Alternative splicing (AS) regulates how a gene can give rise to multiple mature messenger RNAs that differ in sequence and abundance, and after translation possibly regulate different biological processes. The splicing machinery is frequently hijacked by tumors and has been associated with oncogenesis, tumor progression and resistance to anticancer therapy [1, 2]. However, numerous questions about which individual splicing events or splicing factors are involved in these processes remain unanswered.

In the past decade, immune checkpoint blockade (ICB) has revolutionized the treatment of advanced-stage cancers. The success of ICB, however, varies between cancer types. Even within a responsive cancer type, ICB provides durable clinical responses in only a subset of patients. It is well-established that high-tumor mutation burden (TMB) cancer types like lung or skin cancer, which encode numerous neoantigens, are more likely to respond to ICB [3–7]. Nevertheless, some low-TMB lung or skin cancers can also respond well. In other cancer types such as triple-negative breast cancer (TNBC) or prostate cancer, a correlation between TMB and response to ICB is absent [8]. Since T cells require tumor antigens presented by major histocompatibility complex (MHC) class I molecules prior to engage in tumor killing, a key question is how low-TMB tumors succeed in priming T cells to antitumor cytotoxicity. One possibility is that they contain tumor antigens that are not caused by somatic mutations. Indeed, when analyzing tumor proteomes, Khales *et al.* observed that 68% of tumors contain at least one antigen derived from a tumor-specific alternative splicing event (ASE), while only 30% of tumors contain an antigen derived from somatic mutations [9]. For instance, several intron retention (IR) events have been described to give rise to peptide sequences presented by MHC-I in cancer cell lines [10]. By modulating expression of splice regulators in a tumor cell line, numerous tumor antigens were identified using bulk RNA sequencing (bulk RNAseq) and shown to trigger endogenous T-cell responses in mice [11]. Finally, by comparing expression data between tumors and healthy corresponding tissues, tumor-specific splicing junctions that were shared across patients have been identified. Many of these gave rise to MHC-I presented peptides recognized by tumor-infiltrating CD8^+^ T cells or peripheral blood mononuclear cells (PBMCs) [12]. Overall, this illustrates the potential value of splicing-induced antigens during tumor progression and anticancer immunotherapy.

Nevertheless, studies assessing which ASEs contribute to tumor antigenicity have been hampered by technological challenges. Since ASEs are frequent and highly cell type-specific, it is not only important to verify their presentation by MHC-I but also to confirm their unique expression in cancer cells versus other (stromal) cell types. In contrast to bulk RNAseq, single-cell data are ideal to explore this cancer cell-specific expression, as stromal cell types within the same tumor can be considered an “intrinsic” normal tissue reference. Amongst the available single-cell technologies, the droplet-based platform commercialized by 10x Genomics is by far the most popular technology thanks to its scalability and affordability. However, investigating AS in 10x Genomics data is challenging, mainly because it generates short-read single-cell data with biased read coverage. For this reason, the vast majority of established single-cell AS methods, such as BRIE 1&2 [13, 14], SCATS [15], VALERIE [16], Expedition [17], MARVEL [18], and scASfind [19], which were originally designed for Smart-seq2 data, are not suited for droplet-based single-cell technologies. Moreover, most of these pipelines investigate only specific types of ASEs, usually neglecting alternative first and last exon events, or rely only on predefined ASE structures without integrating any *de novo* detection function. Other AS methods, such as SpliZ [20] and MARVEL-AnnotateSJ.10x [18], do not estimate the frequency of each splicing isoform per individual cell, thereby complicating downstream statistical analyses. A long-read sequencing-based single-cell technology obviously alleviates all these challenges, but currently its applications are limited due to high cost [21, 22]. Hence, a comprehensive and effective method for the characterization of AS in scRNAseq data is lacking.

Here, we characterize AS at single-cell level in 42 patients with early BC prior to and during ICB. Using a novel customized bioinformatics approach, we detect and quantify thousands of ASEs specific for cancer cells and various stromal cell types, including T cells. Among the cancer cell-specific ASEs, we identify several potential tumor antigens. We confirm their association with antitumor immune activity and demonstrate how their aggregated load correlates with response to ICB in BC. Finally, we also demonstrate a role for these ASEs during clinical response to ICB in other cancer types.

## Materials and methods

### Study cohort

We analyzed BC data from the clinical trial NCT03197389 described by Bassez *et al.* [23]. The study was approved by the Ethics Committee UZ/KU Leuven (S60100) and all patients provided written consent. Briefly, the study included 54 patients with early operable newly diagnosed stage II primary invasive carcinoma of the breast that was histologically confirmed as estrogen receptor-negative (ER^−^) and progesterone receptor-negative (PR^−^) or ER^+^ BC with a primary tumor size > 1 cm. We merged two cohorts of this clinical trial, including patients scheduled for upfront surgery treatment (*n* = 39), and patients receiving prior neoadjuvant chemotherapy (*n* = 15). A single dose of 200 mg pembrolizumab (Keytruda^®^ or anti-PD1) was delivered prior to surgery in a window-of-opportunity setting, and fresh tumor tissue was collected from these patients before (by needle biopsy) and 7–15 (9 ± 2) days after (by resection) pembrolizumab treatment. In 42 of these samples, 10x Genomics 5′ gene expression profiling was performed on the single-cell suspensions, with 5000 loaded cells for each sample, followed by sequencing on an Illumina NextSeq or/and NovaSeq6000. Patient information was listed in Supplementary Table S1. In Bassez *et al.*, major cell types were identified according to gene expression data. The six most abundant cell types were T cells (*n* = 71 623 cells), cancer cells (*n* = 65 885), fibroblasts (*n* = 37 439), myeloid cells (*n* = 23 146), B cells (*n* = 13 995), and endothelial cells (*n* = 12 464).

### Comparison of read coverage in 5′- and 3′-scRNAseq data

We analyzed five random tissue samples from the above-mentioned clinical trial for which sufficient cells after dissociated were available to subject them to both 5′- and 3′-scRNAseq. We selected protein-coding genes with gene length ≥1000 bps and a “transcript support level” of 1 (i.e. strong evidence) based on Ensembl v93. To estimate the proportion of gene regions with good coverage, exonic regions were separated into 100 bins of equal length and the number of reads per bin were counted. We further selected highly expressed genes based on the average count per bin being ≥5 in both paired 5′- and 3′-data and defined a bin with “good coverage” if it had counts ≥20% of the average across all bins. All highly expressed genes were stratified into three groups based on their gene length. For each bin and for both 5′- and 3′-scRNAseq data, the proportion of genes with good coverage per gene length-group was plotted for each sample and for the five samples merged.

### Detection of AS in 10× 5′-scRNAseq data using JAseC

To detect and quantify ASEs in scRNAseq data, we developed a bioinformatics algorithm referred to as Junction-based Alternative splicing event Counter or “JAseC,” encoded in Julia language (using BioJulia series packages available at https://github.com/BioJulia). Briefly, JAseC can detect and quantify seven AS types and in doing so relies on detecting reads that span a junction between different exons (exon–exon junction reads). In contrast to other methods that also use junction-reads to detect ASEs, JAseC does not consider reads mapping entirely within the alternative exon. It is only when detecting IR events that JAseC also considers reads covering exon–intron boundaries (whereby the intron is the retained sequence that functions as an exon in the inclusion isoform). The advantage of focusing only on exon–exon junction reads is that this allows processing biased read coverage data, such as 5′-scRNAseq data in which read counts gradually decrease from the 5′ to 3′ end. An additional advantage of only considering junctions reads is that JAseC can also detect novel ASEs. When classifying detected junction-reads into one of the seven AS subtypes, JAseC considers known or assembled transcriptome annotations, assigning a splicing type, the affected gene and translational changes to each ASE. Novel ASEs are classified as “unknown.”

More specifically, for most AS types [except for IR and mutually exclusive exons (MXE)] JAseC starts from bam files of the 10x Genomics 5′-scRNAseq data, which are mapped to the GRCh38 human reference genome by 10x Genomics Cell Ranger (v3.1.0) [24]. First, reads with the same UMI are merged before extracting counts and positions of junction-reads from the files. To subsequently detect ASEs, junctions supported by at least 5 or 10 reads (depending on the type of analysis) are grouped based on their splice position. In an ideal situation, an ASE involves an exclusion junction for which one or both splice positions are shared with a splice position from an inclusion junction. For instance, in the case of a cassette exon (CE) both inclusion junctions need to overlap, while in the case of an A5SS or A3SS only one inclusion junction needs to overlap. Sometimes, >2 inclusion junctions share the same splice position with an exclusion junction. In these cases, to prevent double counting, JAseC considers only the top two junctions with the highest read counts for each splice position. To annotate the detected ASEs, junctions and their positions are aligned to exon boundaries. First, JAseC uses Ensembl v93 and then it uses a *de novo* assembled transcript annotation file generated by StringTie (v1.3.4d) [25]. For each ASE, the information of the affected genes, the type of ASE and the effect on the protein sequence are deduced based on annotated transcript structures. ASEs that cannot be linked to annotated transcripts are designated as unclassified (UN). Novel ASEs detected in the immunoglobulin gene regions, T-cell receptor gene regions, and human leukocyte antigen (HLA) genes are ignored because they might be caused by DNA recombination or mapping errors. Additionally, we only considered defined MXEs and IRs using known and assembled transcriptome annotation, as this junction-read-based method is not suitable for these types of ASEs.

After identifying ASEs, the inclusion and exclusion reads of each ASE are counted per cell based on 10x Genomics cell barcodes (UMIs). For the downstream analysis of alternative first exons (AFEs) and alternative last exons (ALEs), proximal and distal isoform junctions are annotated as inclusion and exclusion reads, respectively. JAseC incorporates specific strategies to accommodate the biased read coverage in 5′-scRNAseq data, including exon–exon junction-based ASE detection, minimum read thresholds for both exclusion and inclusion junctions (e.g. ≥10 or ≥5 reads) and tailored approaches for counting inclusion reads. Specifically, for CEs, we chose the inclusion junction with the largest number of reads. For IR, the inclusion read counts are based on the nonjunction-reads that overlap with two splicing positions, whereby the maximum number of observed reads is considered. These approaches avoid unreliable “length normalization” under uneven read coverage, maximize the use of junctions equidistant from the 5′-end of transcripts for both inclusion and exclusion isoform quantifications to neutralize the effects of coverage bias, and produce clean count pairs compatible with downstream binomial model analysis (see further).

Next, the Percent-Spliced-In (PSI) value is calculated by *I*/(*I* + *E*), whereby *I* and *E* are the inclusion and exclusion read counts. The 95% confidence interval of a PSI is calculated using the *binom.test* function in R. The proportion of the inclusion or exclusion alternative isoforms is PSI or 1-PSI, respectively. Importantly, since the junction-read counts identified by JAseC follow a binomial distribution, they are well-suited for exact statistical models when analyzing AS within specific cell groups where read coverage is often limited. For instance, these read counts are compatible with a variety of tools, such as generalized linear models (GLMs), which are widely used in R. In addition, JAseC can be applied to bulk RNAseq data with minimal adaptation (by skipping the UMI unification step), enabling cross-analysis of scRNAseq and bulk RNAseq data. A comparison of the key features of JAseC with other methods characterizing AS in single-cell data is summarized in Supplementary Table S13. This demonstrates that there are currently no other tools capable of detecting ASEs to a similar extent as JAseC.

### AS-based PCA and tSNE analysis

This analysis was conducted across six major cell types. For each cell type, per sample, and per ASE, the inclusion and exclusion read counts across all cells were aggregated. ASEs were further filtered based on the following criteria: (i) the total number of junction-reads per sample should be ≥1 million, (ii) the total number of junction-reads per sample per cell type should be ≥5000, and (iii) the junction-read should be detected in ≥80% of samples within the compared cell types. For each ASE, we modeled a prior PSI distribution using a beta-binomial framework, which enabled us to either impute missing values or refine PSI estimates through a Bayesian approach, especially in cases with limited inclusion or exclusion counts. Specifically, for an ASE*_i_*, we assume that the inherent PSI value follows a distribution of $Beta( {{a_i},{b_i}} )$ in each sample and each cell type comparison, such that the observed inclusion read number ${I_{i,c,s}}$ and exclusion read number ${E_{i,c,s}}$ in cell type *c* and sample *s* follow a beta-binomial distribution. We estimate parameter ${a_i}$ and ${b_i}$ using the R package VGAM (v1.1–4) [26]. This $Beta( {{a_i},{b_i}} )$ was regarded as a prior probability for ASE_*i*_. According to the Bayesian inference, the posterior probability of a given sample-cell type combination is $Beta( {{a_i} + \;{I_{i,c,s}},{b_i} + {E_{i,c,s}}} )$. We used its median as an inferred PSI value, which was calculated by the Distances.jl package (v0.10.0) in Julia (https://github.com/JuliaStats/Distances.jl). Based on the inferred PSI values, principal component analysis (PCA) was performed using the MultivariateStats.jl package (v0.8.0) (https://github.com/JuliaStats/MultivariateStats.jl) in Julia, and t-distributed stochastic neighbor embedding (tSNE) was performed using the scikit-learn package (v0.21.2) in Python [27]. For the permutation analysis (Supplementary Fig. S1D) in order to remove splicing ratio information from the data, while keeping the expression (coverage) information, ${I_{i,c,s}}$ and ${E_{i,c,s}}$ were randomly reassigned according to $Beta( {{a_i},{b_i}} )$ under the condition that ${I_{i,c,s}}$+${E_{i,c,s}}$ were kept unchanged.

### Detection of cell type or sample-specific ASEs

To identify ASEs specific to a given cell type compared to others, or between two sample groups, such as estrogen receptor-positive BC (ER^+^ BC) versus TNBC, T-cell expanders (Es) versus nonexpanders (NEs), or pre- versus on-treatment samples, the following analysis was performed (here, we use the comparison between Es versus NEs as an example). We first selected ASEs with sufficient read coverage, requiring that the total exclusion and inclusion read number across all samples, as well as the total number of junction-reads between E and NEs should be ≥10. An additional filter was applied to ensure that neither alternative isoform represented splicing noise. Specifically, assuming *H_X_* and *L_X_* represent the upper and lower bounds of the 95% confidence interval of PSI values in group *X*, ASEs were required to meet all four of the following conditions:

1. *L_E_* > 0.05 or *L_NE_* > 0.05; and
2. *H_E_* < 0.95 or *H_NE_* < 0.95; and
3. *L_E_* > 0.05 or *H_E_* < 0.95; and
4. *L_NE_* > 0.05 or *H_NE_* < 0.95.

For the ASEs that passed both filters, we refer to them as “detected ASEs.” A binomial-based GLM in R language is then applied using the inclusion and exclusion read numbers per sample, and likelihood ratio tests to estimate the importance of the group variable (E versus NE), followed by Benjamini–Hochberg multiple test correction. ASEs with FDR < 0.01 and |ΔPSI| > 0.2 are considered differentially spliced (i.e. “specific” ASEs).

**Figure 1.**
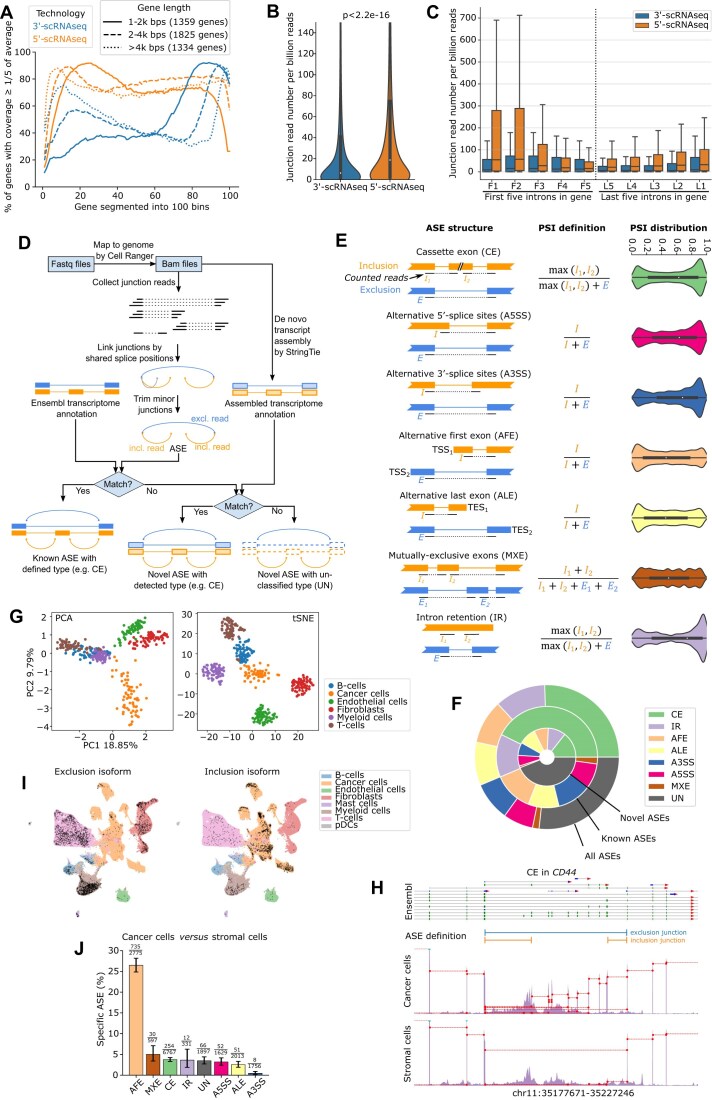
Exploring AS in breast tumors. (**A**) Percentage of genes with good read coverage (count per bin ≥ 1/5 of the average) in different gene regions (100 equal-length bins), based on merged 3′- and 5′-scRNAseq data (see Materials and methods). Genes are stratified by length, as indicated in the legend panel. The five samples were derived from BC biopsies (see Materials and methods). (**B**) Density distribution of junction-reads in 3′- or 5′-scRNAseq data. Junction-reads among 187 474 introns were counted based on the major isoform of coding genes using 3′- or 5′-scRNAseq data of five BC biopsies. Significance between 3′- and 5′-scRNAseq data was assessed using the Wilcoxon test. (**C**) Density distribution of intronic junction-reads located in the first five (F1–F5) and last five (L1–L5) introns of a representative isoform of a coding gene in the 3′- or 5′-scRNAseq data. Box-plots extend from the first quartile (Q1) to the third quartile (Q3) of the data, with a line at the median. The whiskers show the range of the data except for the outliers beyond 1.5× the inter-quartile range. (**D**) Schematic representation of ASE detection by JAseC, taking the CE as an example. (**E**) Schematic overview of the ASE structures (left), PSI definition (middle), and the PSI distribution (right) for each AS type. “TSS”: transcription start site; “TES”: transcription end site. (**F**) Proportion of different AS types among all detected ASEs (outer circle), detected known ASEs (middle circle), and detected unknown ASEs (inner circle). (**G**) PCA (left panel) and tSNE (right panel) plots based on PSI values of ASEs across major cell type and tumor sample. A total of 4247 ASEs with sufficient coverage across all cell types and samples were considered. Each dot represents a cell type from one tumor sample. (**H**) Read coverage of ASEs detected in *CD44*, shown separately for cancer cells and stromal cells in pre-treatment BC samples. The top panel shows Ensembl annotation, with blue, green, and gray lines representing the untranslated region (UTR)/noncoding exons, coding sequence (CDS), and introns, respectively. Red triangles indicate transcript direction. Lower panels show read coverage, stratified by cancer cells versus stromal cells. Junction-reads are drawn in red dashed lines unless their height is <1% of the *y*-axis. (**I**) UMAP showing cells that express either the normal *CD44* isoform (exclusion isoform) or the tumor-specific *CD44v* isoform (inclusion isoform). Cell types are colored according to Bassez *et al.* [23]. Black dots indicate cells expressing the respective isoforms. (**J**) Percentages of ASEs specific to cancer cells versus stromal cells grouped by AS type. The number of specific ASEs (numerator) and total detected ASEs (denominator) is labeled above each bar. Error bars show 95% confidence intervals.

Other analyses compared ASE differences between two groups of cells across samples, such as the comparison of cancer versus stromal cells in Fig. 1J. Here, ASEs were first filtered based on the merged read counts and confidence intervals between two groups of cells according to similar criteria. For ASEs that passed filtering, two binomial-based GLMs were constructed using the inclusion and exclusion junction-read counts in each sample for the cell type comparison being tested: *H*_1_: *(I*_count_, *E*_count_*)∼*sample + group, *H*_0_: *(I*_count_, *E*_count_*)∼*sample. The goodness of fit comparison between the two models was performed by likelihood ratio test, followed by Benjamini–Hochberg adjustment. Specifically, to detect cell type-specific ASEs, each major cell type (group = ‘A’) was compared against the merged set of all other cell types (group = ‘B’). For analyses of subtype-specific ASEs within T- or B cells, independent tests were performed for all pairwise comparisons.

### Functional enrichment analysis

Functional enrichment analysis was performed to identify pathways, gene ontologies, or “hallmarks gene sets” [28] that were overrepresented in our lists of differentially spliced genes. For each gene list tested, we simultaneously performed enrichment analysis on five gene set categories, including “Hallmarks,” “KEGG,” and three “gene ontology” sets (biological process, molecular function, and cellular component). All gene sets were downloaded from Molecular Signatures Database (MSigDB) [29]. For each gene set in any of the five categories, one-tailed Fisher’s exact tests were used to assess enrichment for the gene list tested (i.e. genes with specific ASEs) against the background gene list (i.e. genes only containing nonspecific ASEs defined by the same coverage cutoffs), followed by Benjamini–Hochberg multiple test correction (cutoff: FDR < 0.05). These analyses were further corrected for gene expression bias. Briefly, all test and background genes were grouped into 10 bins according to the expression of each gene. Down-sampling of genes in each bin was performed for background genes until an equal distribution of test and background genes in each bin was obtained. The resulting test and background genes from each bin were subsequently assembled as a background and test gene list.

### RNAseq analysis in cell lines with *NOVA1* or *ESRP1* knock-down, or *ESRP1* overexpression

For public *ESRP1* knock-down and overexpression data, bulk RNAseq data from PC3 cells treated with or without siESRP1 were obtained from GEO (GSE139962). Bulk RNAseq data from the *ESRP1* stably overexpressing cell line ESRP1-SKOV3 and its control group, EV-SKOV3, were downloaded from GEO (GSE133841). Both datasets include three biological replicates per condition.

For the in-house *ESRP1* knockdown experiments, MCF7 BC cells were cultured in Dulbecco’s Modified Eagle Medium (DMEM) supplemented with 10% fetal bovine serum (FBS) under standard conditions according to American Type Culture Collection (ATCC) guidelines. Short hairpin RNAs (shRNAs) targeting *ESRP1* (TRCN0000149820 and TRCN0000147725) were obtained from the RNAi Consortium (TRC). Lentiviral particles were generated by co-transfecting HEK293 T-cells with the shRNA plasmids using GeneJuice Transfection Reagent (Novagen), following the TRC protocol. These lentiviral particles were then used to transduce MCF7 cells. After transduction, cells were selected with puromycin (1 µg/ml, InvivoGen) for 3 days to establish stable *ESRP1* knockdown lines. As control, cells were transduced with lentivirus carrying an empty vector. For *NOVA1* knockdown, BT-549 cells were transduced using a similar protocol, with shRNA construct TRCN0000001089. For *ESRP1* overexpression, MCF7 cells were similarly cultured and transduced with lentiviral particles generated using an overexpression construct pLVX-ESRP1-mCherry-Puro obtained from Novopro. For all experimental conditions (two replicates for each), total RNA was then extracted, and libraries were prepared for 2 × 150 bp paired-end polyA-selected RNA sequencing on the Illumina NovaSeq6000 platform.

In the subsequent bioinformatics analysis, FASTQ files were mapped to the human genome (GRCh38) using STAR (v2.5.2a). Based on ASEs detected in scRNAseq BC data, inclusion and exclusion reads for each ASE were counted by bulk RNAseq using a modified version of JAseC (which does not consider UMIs and cell barcodes), and the PSI values were calculated by summing read counts across all three experimental replicates. For each replicate, ASEs were filtered by requiring ≥10 junction-reads in both treatment and control samples, and ≥5 total junction-reads supporting either alternative isoform. Differential splicing between treatment and control was assessed using Fisher’s exact test under cutoff *P *< 0.05 and |ΔPSI|>0.1. To reduce noise, only ASEs that were differentially spliced in at least two replicates with consistent directional changes were included in the downstream correlation analysis with E versus NE splicing changes from the BC scRNAseq data.

### Analysis of CLIP-seq data targeting *ESRP1* in the SGC7901 gastric cancer cell line

CLIP-seq fastq files were downloaded from GSE233931 [30]. Read adaptors were trimmed using Fastp, followed by mapping to the human genome (GRCh38) with STAR. Crosslink sites were called using PureCLIP at a score cutoff of 1, and the two replicates were merged. An ASE was considered as *ESRP1*-bound, if any crosslink site was detected within ± 50 bps of its splice sites. For this analysis AFEs were excluded, as they are more likely to be regulated by transcription factors.

### Splicing distance index for subtypes of T cells

We created a splicing distance index to quantify the similarity between two T-cell subtypes at the AS level. For ASEs specific for one of the T-cell subtypes, the PSI for each subtype was linear-scaled between 0.01 and 0.99 and transformed by logit function. The splicing distance between two subtypes was calculated using the Euclidean distance of the transformed PSIs based on ASEs expressed in both subtypes.

### Parallel single-tube long-fragment-read sequencing and data analysis

Single-tube long-fragment-read (stLFR) libraries were built based on four full-length cDNA samples isolated from the 10x Genomics 5′-scRNAseq platform. cDNAs were derived from pre-treatment tumors (of patient number: 7, 10, 12, and 20), quantified, and polymerase chain reaction (PCR) amplified. Subsequently, the stLFR procedure was followed as reported [31–34]. Briefly, with the addition of adapters, amplified cDNA products were circularized via ligation and then underwent Rolling Circle Amplification (RCA). Subsequent stLFR libraries were prepared using the regular stLFR kit (MGI Tech), and the final libraries were sequenced on DNBSEQ platform from MGI Tech. stLFR raw reads were first assembled based on the stLFR bead barcodes according to the IterCluster algorithm [35] and then mapped to the human genome (GRCh38) using STAR. Next, all technical replicates of each sample were merged, and reads with the same UMI were further linked using a custom Julia script. We harvested a total of 33.4 million UMI-containing assembled reads (i.e. “stLFR reads”). Using the ASE structures and UMI-to-cell type annotation from the scRNAseq data, we counted the inclusion and exclusion stLFR reads and calculated PSI values per cell type and sample and used this to validate the 5′-scRNAseq data. To validate ASE structures using stLFR long reads, we utilized 200 million stLFR reads, including those with and without UMI tags. An ASE was considered validated if stLFR reads covered all the exons involved in the AS event and this for both isoforms. The ASEs belonging to the unclassified AS subtype (UN) were not included in this analysis.

### Single-cell ATAC-sequencing data analysis

We performed 10x Genomics Chromium Single Cell ATAC on the pre- and on-treatment samples of 12 BC patients (Supplementary Table S6). Fastq files were first aligned to the GRCh38 genome by Cell Ranger ATAC (v1.2.0) [36]. ATAC peaks of each sample were called by MACS2 (v2.1.2) [37], and consensus peaks were generated using https://github.com/aertslab/iterative_peak_filtering, which implements an iterative peak filtering method based on [38]. The major cell types were identified using pycisTopic (v1.0) [39]. Based on the consensus peaks, count-per-million (CPM) per sample and per cell type were calculated using a custom Julia package. For each alternative promoter containing an AFE, if there are ATAC consensus peaks in its promoter region (−2k bp to +500 bp of TSS), the absolute promoter accessibility was estimated by the CPM of this consensus peak (or of the peak with the highest mean CPM if there is more than one peak in the promoter region). For the AFEs whose two promoter regions partially overlapped, the ambiguous peaks were assigned to the nearest alternative promoter. The relative promoter accessibility was defined by log2 fold changes of proximal versus distal peak CPM (offset 0.1).

### Splicing similarity score analysis in T cells

We first identified ASEs specific for a T-cell subtype by performing pairwise comparisons of T-cell subtypes. For instance, in CD8^+^ T cells we compared naïve versus effector-memory cells, effector-memory versus exhausted cells, as well as naïve versus exhausted cells, to obtain 17, 45, and 25 ASEs with a cutoff *p*_GLM _< 0.05 and |ΔPSI |> 0.2. For each pairwise comparison between T-cell subtypes A versus B, we defined the list of specific ASEs as *L* and estimated the PSI for each ASE based on the combined reads of all T-cell subtypes in each tumor sample *t*. This was defined as${\mathrm{\;PS}}{{\mathrm{I}}_{l,t}}$. Supposing ${\mathrm{PS}}{{\mathrm{I}}_{l,A}} < {\mathrm{PS}}{{\mathrm{I}}_{l,B}}$, we defined the similarity score of *l* and *t* as:


\begin{eqnarray*}
{S_{l,t}} = \left\{ {\begin{array}{@{}*{1}{c}@{}} {0,\quad if\quad {\mathrm{PS}}{{\mathrm{I}}_{l,t}} \le {\mathrm{PS}}{{\mathrm{I}}_{l,A}}}\\ {1,\quad if \quad{\mathrm{PS}}{{\mathrm{I}}_{l,t}} \ge {\mathrm{PS}}{{\mathrm{I}}_{l,B}}}\\ {\frac{{{\mathrm{PS}}{{\mathrm{I}}_{l,t}} - {\mathrm{PS}}{{\mathrm{I}}_{l,A}}}}{{{\mathrm{PS}}{{\mathrm{I}}_{l,B}} - {\mathrm{PS}}{{\mathrm{I}}_{l,A}}}},\quad if \quad{\mathrm{PS}}{{\mathrm{I}}_{l,A}} < {\mathrm{PS}}{{\mathrm{I}}_{l,t}} < {\mathrm{PS}}{{\mathrm{I}}_{l,B}}} \end{array}} \right.
\end{eqnarray*}

If ${\mathrm{PS}}{{\mathrm{I}}_{l,A}} > {\mathrm{PS}}{{\mathrm{I}}_{l,B}},$ the same formula applies, but A and B are switched. The similarity score for a sample, i.e. ${S_t}$, was calculated as the mean value of ${S_{l,t}}$, where *l* is the ASE in *L* and has at least five reads in the T cells of sample *t*. The samples with insufficient reads in T cells, i.e. samples for which more than one-third of ASEs in *L* had <5 reads per sample, were excluded in the results of Fig. 5J and Supplementary Fig. S5D.

### Identification of tumor-specific atypical ASEs

We defined a new threshold to identify atypical ASEs since we were specifically interested in identifying AS-derived tumor antigens. We reasoned that a strict cutoff would be needed, especially for normal tissue, as even low expression levels of the isoform in one normal tissue could lead to antigen presentation, thereby triggering immune tolerance. We therefore selected ASEs in each cell type if both the inclusion and exclusion read numbers were ≥5, and if neither of both isoforms could be considered as splicing noise. For the latter, we examined the upper and lower bounds of the 95% confidence interval of the PSI and retained only those ASEs for which these values were between 5% and 95%. PolyA-based bulk RNAseq data of adult normal tissues were downloaded from ENCODE (*n* = 48 samples from 19 different tissues, testis was excluded; Supplementary Table S8). ASEs were considered tumor-specific atypical ASEs if the proportion of either alternative isoform (i.e. PSI for the inclusion isoform, or 1-PSI for the exclusion isoform) was at least 10 times higher than the mean proportion in normal tissues, and the lower bound of its confidence interval was at least four times higher than any proportion value in individual normal tissues.

### Atypical ASEs giving rise to tumor antigens in breast cancer

To detect potential tumor antigens amongst atypical ASEs, we required ASEs to be undetectable in any of the 113 normal breast tissue bulk RNAseq data downloaded from TCGA. Additionally, the atypical isoform had to be largely absent in tumor stromal cells, which we also considered as normal breast tissue reference data. Specifically, we required the proportion of atypical isoform (PSI or 1-PSI) observed in cancer cells to be at least five times higher than the mean proportion observed in stromal cells, and the lower bound of its 95% confidence interval to be at least twice as high as that in stromal cells in any individual patient. This criterion is slightly less restrictive than that used to define tumor-specific atypical ASEs, allowing for potential mixing of cancer and stromal cells in the scRNAseq data due to doublet formation or clustering errors. Candidate antigenic ASEs included CEs, A5SS, A3SS, and IRs located within coding regions according to their Ensembl annotation. We estimated their potential to undergo nonsense-mediated decay (NMD) based on the “50nt-rule,” which mostly defines an isoform containing a premature stop codon that is downstream of the ASE but not in the last exon of the gene to undergo NMD [40]. If ASEs were predicted to undergo NMD, they were not considered as potential antigens. To estimate the MHC-binding potential of atypical isoform products, we translated peptides of both the atypical and normal isoforms, and extracted unique 8–11 mer amino acid fragments, excluding any fragments existing in the human normal protein sequence according to UniProt database [41]. These fragments were then analyzed using netMHCpan (v4.1) according to the recommended strong binder cutoff of “%rank < 0.5.” Patient HLA allele types were inferred using OptiType (v1.3.5) [42]. Binding predictions were performed for each of the six HLA alleles detected per patient, and the strongest binder (i.e. the one with the lowest “%rank”) was used for downstream analysis. Atypical isoforms predicted not to undergo NMD and containing a unique peptide N-mer predicted as strong MHC binder were considered potential tumor antigens. Isoform abundance in each sample was calculated by multiplying the isoform proportion (PSI or 1-PSI) with expression (represented by CPM) of the respective gene in cancer cells. The ASE-derived tumor antigen burden (ATB) for each sample was then computed as the sum of the isoform abundances of all potential tumor antigens with strong MHC-binding affinity.

### Validation of tumor-specific atypical isoform peptides using proteomics data

Raw label-free proteomic mass spectrometry (MS) data from a nanoscale LC hyphenated LTQ-Orbitrap-XL MS/MS system for a total of 125 TNBC tissue samples were downloaded from ProteomeXchange (ID: PXD000260) and converted to MGF files using ProteoWizard’s msConvert [43]. A searching database was created using the UniProt human reference proteome (taxonomy ID 9606, containing 20 375 reviewed protein entries), extended with a set of common contaminants and the predicted sequences of the tumor-specific atypical ASEs. A target-decoy strategy with reverse decoys was used to allow false discovery rate (FDR) calculations. Search engines Comet [44] and MS-GF+ [45] were run through SearchGUI (v4.3.11) [46] with the following parameters: a precursor *m/z* tolerance of 7 ppm, a fragment *m/z* tolerance of 0.4 Da, an FDR of 5%, the carbamidomethylation of cysteines as fixed modification, and N-terminal acetylation and methionine oxidation as variable modifications. The search results were then combined and interpreted using PeptideShaker (v3.0.11) [47].

### Analyses on “The Cancer Genome Atlas (TCGA)” bulk RNAseq data

For BC, bam files of 1099 primary BC tumors and 113 normal breast tissues were downloaded from TCGA (https://www.cancer.gov/tcga). Sample TCGA-A2-A0EX-01A was excluded because its bam file was found to be incomplete. The inclusion and exclusion junction-reads of the 34 ASE-induced tumor antigens detected in the scRNAseq BC data were counted in these bam files using the Julia packages XAM.jl (v0.2.7) and GenomicFeatures.jl (v2.0.4). The HLA genotype of each sample and MHC-binding prediction of each of the 34 ASEs were performed as described (see above). ATB was also calculated for each sample, as described, except that gene expression was represented by fragments per kilobase per million mapped fragments (FPKM) instead of CPM. Gene FPKM values were retrieved from the TCGA website. The gene lists of the signature used to estimate immune activation and other immune states in TCGA tumors are listed in Supplementary Table S10. Cytolytic activity [48] and the immuno-predictive score (IMPRES) [49] were calculated as described. Other single gene signatures were using the log_2_(FPKM + 1) and were calculated by the R package GSVA (v1.34.0) [50] under parameter kcdf = ‘Poisson’. Partial Spearman’s correlations were calculated by the R package PResiduals (v1.0) [51]. To perform gene set enrichment analysis (GSEA) on genes correlated with ATB, genes were ranked based on their –log10 of *P*-values in the corresponding Spearman’s correlation test, multiplied by the sign of the correlation coefficient.

In the pan-cancer analysis, gene expression counts and bam files of 6557 samples from 16 solid cancer types were downloaded from TCGA, and the reads involving the inclusion and exclusion junctions of *PPP1CB*-TAI were extracted and counted. To test splicing differences involving *PPP1CB*-TAI between tumors and normal tissues in each type of cancer, a beta-binomial GLM based on inclusion and exclusion read counts was built using the R package VGAM (v1.1) [52]. To determine the correlations between *PPP1CB*-TAI and bulk RNAseq immune signatures, a beta-binomial GLM was built per signature using the R package VGAM (v1.1), with inclusion and exclusion read counts as the dependent variable, and immune signatures and cancer type as independent variables. The significance of the overall effect was estimated by the likelihood ratio test compared to the simplified model without the signature variable being included.

### PCR validation of antigenic atypical ASEs

Full-length cDNA was generated from the tumor biopsy of patient #7, #10, #11, #14, #20, #31, and #29 to validate expression of antigenic ASEs, as well as normal tissue as a negative control PBMC or normal tissues adjacent to head and neck squamous cell carcinoma) using SuperScript IV reverse transcriptase and oligo(dT)20 (Invitrogen #18091050). Oligonucleotides were designed to bind the constitutive exons before and after the respective ASEs, making sure both the short product resulting from the exclusion exon and the long product resulting from the inclusion exon were detectable (≥100 bp) and separatable (difference ≥ 35 bp). They were used in combination with the Q5 High-Fidelity master mix (New England Biolabs #M0492S) to amplify the different splice variants.

### Melanoma single-cell data analysis

Melanoma single-cell data have been reported by Pozniak *et al.* [53] (Supplementary Table S12). Fastq files were aligned to the human genome (GRCh38) and pre-processed using Cell Ranger (v3.1.0). Using scanpy (v1.8.2) [54], cells were filtered by requiring 200–6000 genes to be detected per cell of which <25% represented mitochondrial reads. For cell clustering analysis based on gene expression, the top 2000 highly variable genes were selected and normalized, while regressing for cell count numbers, mitochondrial read proportions and cell cycling scores (S and G2M). The top 20 PCs were selected according to the elbow method on the contribution curve and clustering was performed using the Leiden method. The cell type of each cluster was manually identified using known marker genes (Supplementary Fig. S8B and C). For AS analysis, the bam files of all melanoma and BC samples were imputed into JAseC to obtain a uniform definition of all ASEs. Potential tumor antigen detection amongst the detected ASEs was performed as described for BC samples, but this time only melanoma samples were considered.

### Pan-cancer ICB cohort analysis

Bulk RNAseq data of metastatic urothelial cancer (mUC), nonsmall cell lung cancer (NSCLC), and renal cell carcinoma (RCC) were downloaded from EGA (no. EGAD00001006631). Bulk RNAseq data of two malignant melanoma cohorts were downloaded from dbGaP (no. phs000452.v3.p1) and GEO (no. GSE91061). Fastq files were mapped to the human genome (GRCh38) using STAR (v2.5.2a), and the reads of inclusion and exclusion junctions of *PPP1CB*-TAI were counted by custom programming. To explore the effect of *PPP1CB*-TAI on ICB response, a beta-binomial GLM model was built using the R package VGAM (v1.1), with inclusion and exclusion read counts as the dependent variable, and cohorts and ICB response as independent variables. To analyze the effects of both *PPP1CB*-TAI and TMB on ICB response across cohorts, a binomial GLM model was built, with ICB response as the dependent variable, and cohorts, TMB, as well as a logit-transformed *PPP1CB*-TAI proportion (adapted to a 1%–99% range to avoid the infinity problem) as independent variables. For the progression-free survival (PFS) analysis, a Cox proportional-hazards model was preformed using the R package survival (v3.2) [55] with cohort and logit-transformed *PPP1CB*-TAI proportions as independent variables, with or without TMB. The contribution of *PPP1CB*-TAI or TMB was statistically assessed using the likelihood ratio test.

## Results

### AS detection in single-cell data of the breast tumor microenvironment

To assess the feasibility of studying AS in single-cell RNAseq (scRNAseq) data, we explored read coverage in 10x Genomics Single Cell 5′ and 3′ Gene Expression data. In the 5′-scRNAseq data, read coverage was not only confined to the first 95 bps of the gene, which according to the 5′ protocol corresponds to the read length that is directly sequenced. Indeed, we found that for most genes >20% of reads (relative to the average read coverage of this gene) mapped outside of the first 95 bps from their 5′ start site. In contrast, in 3′-scRNAseq data significantly less genes were characterized by high read coverage in regions >95 bps upstream of the 3′ end (Fig. 1A and Supplementary Fig. S1A and B for individual samples or genes). We additionally noticed that in 5′-scRNAseq we were able to recover significantly more reads mapping to >1 exon, i.e. junction-reads mapping across exon–exon boundaries, than in 3′-scRNAseq data. Indeed, when comparing relative abundances of junction-reads on five samples with both 5′- and 3′-scRNAseq data available, the number of junction-reads per billion total reads was three times higher in 5′- than in 3′-scRNAseq data (18.69 versus 6.27, Wilcoxon test, *P *< 2.2e-16). This increase was most pronounced but not limited to the first three exons of genes (Fig. 1B and C). Hence, compared to 3′-scRNAseq, 5′-scRNAseq is more informative to detect ASEs based on junction-reads.

We next analyzed 5′-scRNAseq data in a unique set of tumor biopsies collected from 42 early BC patients during neoadjuvant anti-PD1 (pembrolizumab) treatment. Specifically, 84 paired samples were available, collected prior to and after one dose of pembrolizumab [23] (Supplementary Table S1). To detect and quantify ASEs in these 5′-scRNAseq data, we developed a computational method coined “Junction-based Alternative splicing event Counter” or “JAseC.” JAseC adopts existing bulk RNAseq pipelines that detect reads spanning exon–exon junctions within a gene, while incorporating cellular barcodes and unique molecular identifiers (UMI) inherent to single-cell data (Fig. 1D, see Materials and methods). Specifically, JAseC considers ASEs supported by at least 10 junction-reads, allowing both a reference and alternative AS isoform to be defined. It detects both known (based on v93 Ensembl transcript annotation) and novel (un-annotated) ASEs (see Materials and methods). For instance, when applied to our BC 5′-scRNAseq data, JAseC detected 65 293 ASEs, of which the majority (60%) was novel. JAseC also detected various different types of ASEs (Fig. 1E for visual representation), the majority representing exon-skipping or cassette exon events (CE, including multiple exon-skipping events; 26%) and AFE (i.e. alternative promoter) events but also events involving ALEs, alternative 5′-splice sites (A5SS or alternative donor sites), alternative 3′-splice sites (A3SS or alternative acceptor sites), MXE, IR, and unclassified ASEs (UN) (Fig. 1F). In total, 20 498 ASEs occurred in protein-coding regions, of which 47% are predicted to generate premature stop codons 50 bps before the last exon–exon junction. Therefore, these ASEs are presumed to undergo NMD [40]. Other ASEs were in UTRs (*n* = 26 106) and in transcripts derived from nonprotein coding regions (*n* = 18 698).

Finally, although junction-reads were more scattered throughout genes in 5′- compared to 3′-scRNAseq data, there is still a significant biased read coverage. By considering only junctions covered by at least 10 junction-reads, in addition to several other measures (see Materials and methods), JAseC efficiently handles this bias. Indeed, when comparing the number of ASEs detected in 5′-scRNAseq data as a function of their distance from the 5′ end of each gene, we observed an almost constant distribution along the normalized gene length, especially for medium to highly expressed genes (Supplementary Fig. S1C). Notably, this did not differ from the distribution of ASEs detected in bulk RNAseq data, which unbiasedly detect ASEs along genes.

### An atlas of cell type-specific ASEs in breast tumors

AS is often regulated in a cell type-specific manner [56, 57]. We therefore explored whether ASEs could identify the major cell types annotated previously based on scRNAseq expression (i.e. cancer cells, endothelial cells, fibroblasts, T cells, B cells, and myeloid cells) [23]. We limited this analysis to 4247 ASEs for which the corresponding gene was expressed and had sufficient coverage (≥10 junction-reads) in all patients and major cell types. For each ASE, we calculated the PSI value, which represents the proportion of inclusion or exclusion junction-reads relative to the total number of junction-reads that support each ASE (Fig. 1E). Accordingly, the inclusion and exclusion isoform proportions are represented by PSI and 1-PSI, respectively. PCA and tSNE-based clustering of PSI values per tumor and per annotated cell type revealed several clusters. These all consisted of the same cell type annotated based on scRNAseq expression: in the PCA, the first principal component (PC) separated immune from nonimmune cells, while the second PC separated cancer from other stromal cells (Fig. 1G). To illustrate that these clusters were not driven by expression levels of individual genes, we repeated the same analysis by randomly shuffling inclusion and exclusion read numbers per ASE, while keeping the sum of the reads for each junction constant. This failed to identify obvious cell type patterns in the PCA and tSNE plot, confirming that the original clustering was determined by ASEs (Supplementary Fig. S1D).

Next, given their cell type specificity, we generated an atlas of ASEs specific for cancer cells and each stromal cell type. For each ASE, we applied a binomial regression on the inclusion and exclusion junction-read counts per cell type and per sample, and then applied a FDR < 0.01 and ΔPSI > 0.2 as cutoff to consider an ASE “specific” for a group of samples or cell type. When comparing ASEs from each cell type versus all other cell types and requiring sufficient read coverage (≥10 junction-reads) per group of cells compared (Materials and methods), a total of 2710 ASEs out of 20 106 ASEs considered, were specific to one of the cell types (Supplementary Table S2). When focusing on ASEs specific for cancer versus all stromal cells, we detected 17 765 ASEs with sufficient read coverage in both cancer and stromal cells. Of these,  1208 ASEs in 1044 genes were specific for cancer or stromal cells. Most alternative isoforms (∼73%) were detected both in cancer and stromal cells, but ∼27% were almost uniquely expressed in either cancer or stromal cells (ΔPSI > 99%). Interestingly, we identified a cancer cell-specific ASE in the cell adhesion gene *CD44*, which represents a glycoprotein involved in cell adhesion and migration that provides a textbook example of how AS can generate various isoforms with properties that may have distinct biological effects. The alternative isoform that was uniquely present in cancer cells was represented by the *CD44v3-v10* isoform, which involves a junction spanning exon 5 and 7. This isoform is indeed known to be prevalent in advanced tumors where it drives metastasis [58, 59] (Fig. 1H and I). Overall, this confirms that JAseC can also detect known splice differences.

Among the seven types of ASEs, AFEs most frequently gave rise to ASEs specific for cancer *versus* all stromal cells: up to 26% (735 out of 2775) of AFEs versus ∼3.2% (473 out of 14 517) of non-AFEs (Fig. 1J). Similar observations were done for other cell types (Supplementary Fig. S1E), and this independently of read coverage depth (Supplementary Fig. S1F). Consequently, when comparing tSNE-based clustering of PSI values with AFEs *versus* non-AFEs, we noticed improved clustering of cell types by the former (Supplementary Fig. S1G).

### AS according to breast cancer subtype

**Figure 2.**
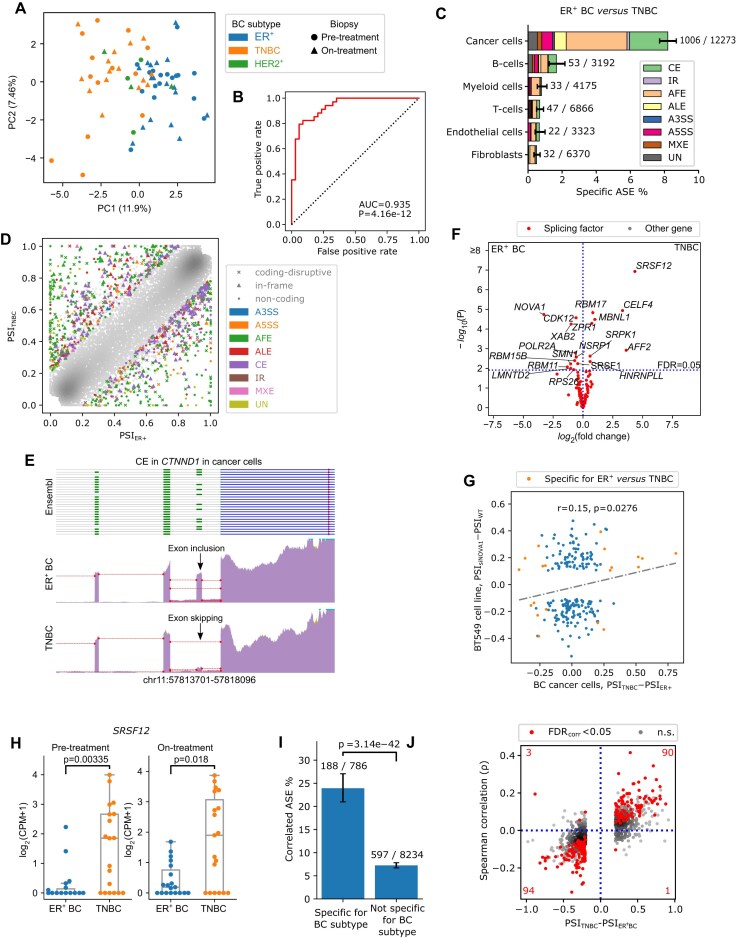
AS according to BC subtypes. (**A**) PCA plot of BC samples based on PSI values from cancer cells. A total of 6602 ASEs with sufficient coverage in cancer cells across all samples were considered. (**B**) Receiver operating characteristic (ROC) curve using the first principal component (PC1) to classify ER^+^ BC versus TNBC subtypes; AUC: area under the curve. (**C**) Proportion of specific ASEs versus all ASEs detected in the pre-treatment samples comparing ER^+^ BC versus TNBC. Color coded by ASE type. The number of specific ASEs and detected ASEs were labeled on the right side of each bar. Error bars show 95% confidence intervals. (**D**) Scatter plots showing the PSI of BC subtype-specific ASEs for ER^+^ BC (*x*-axis) versus TNBC (*y*-axis). Each color represents an AS type and each marker shape represents three deductive effects on translation: (i) AS changes translation initiation or shifts the open-reading frame (“coding-disruptive”), (ii) AS changes the coding region but maintains the open-reading frame (“in-frame”), or (iii) AS has no impact on the protein-CDS (“noncoding”). (**E**) Read coverage of ASEs detected in *CTNND1* in cancer cells from ER^+^ BC versus TNBC pre-treatment samples, plotted in the same style as Fig. 1H. (**F**) Volcano plot showing differentially expressed genes comparing ER^+^ BC versus TNBC. Red dots represent all 111 splicing regulator genes. (**G**) Scatter plots showing the ΔPSI of BC subtype-specific ASEs comparing ER^+^ BC versus TNBC in cancer cells (*x*-axis), against the ΔPSI observed under *NOVA1* knockdown versus controls in the MCF7 cell line (*y*-axis). (**H**) SRSF12 expression in cancer cells of each sample stratified by BC subtype. (**I**) Proportion of ASEs with a significant Spearman’s correlation (FDR < 0.05) between PSI and *SRSF12* expression (CPM) at the single-cell level amongst all cancer cells, stratified by whether ASEs are differentiated between ER^+^ BC and TNBC in cancer cells. Significance between groups was tested using Fisher’s exact test. Error bars show 95% confidence intervals. (**J**) Scatter plot where each dot represents an ASE, showing the PSI difference between TNBC and ER^+^ in cancer cells (*x*-axis), and the Spearman’s correlation (ρ) between its PSI and *SRSF12* expression at the single-cell level across all cancer cells (*y*-axis). ASEs with significant correlations (Spearman’s correlation tests followed by Benjamini–Hochberg correction, FDR < 0.05) are highlighted in red. The number of red dots in each quadrant is labeled in red in the corresponding corner.

Next, we characterized cancer cell-specific ASEs in more detail. PCA of PSI values for cancer cell-specific ASEs revealed separation between ER^+^ and TNBC by the first PC (AUC = 0.94, *P *= 4.16e-12; Fig. 2A and B). Binomial regression identified 1006 ASEs (or 8% of all detected ASEs in this comparison) that were specific for ER^+^ versus TNBC in cancer cells (Fig. 2C and D, and Supplementary Table S3). For instance, we noticed that the penultimate exon of *catenin delta 1* (*CTNND1*), a tumor suppressor often linked with the development and progression of BC [60, 61], was more frequently skipped in TNBC than ER^+^ BC (Fig. 2E). In total, up to 894 genes were affected by BC subtype-specific ASEs and were enriched in Gene Ontology (GO) molecular functions “cell adhesion molecule binding” (*FLNA, CTNND1, PTK2*) and “anchoring junction” (*AKT1, PIK3R1, CTNND1*), and biological process “cytoskeleton organization” (*NCOR1, CYLD, NUMA1*) (Supplementary Fig. S2A). These functions relate to epithelial–mesenchymal transition, which is linked to metastasis and treatment resistance in BC and is more prominently activated in TNBC [62, 63].

To explore which potential factors mediate these subtype-specific ASEs, we assessed expression of genes regulating the RNA splicing machinery (*n* = 111) between both BC subtypes. This revealed 17 differentially expressed splicing factors (Fig. 2F). The gene most highly expressed in ER^+^ BC was *NOVA1*. We further analyzed expression of splicing regulators in “The Cancer Genome Atlas (TCGA)” (*n* = 1099) and confirmed that *NOVA1* was specifically upregulated in ER^+^ BC (Wilcoxon test, *P *= 2.68e-36; Supplementary Fig. S2B and C). Next, we performed small interfering RNA (siRNA)-mediated knock-down of *NOVA1* in the BC cell line MCF7, followed by bulk RNAseq, and found that ΔPSI values in cancer cells observed between ER^+^ BC versus TNBC showed a significant positive correlation with those obtained from wild-type versus *NOVA1* knockdown conditions (Pearson correlation: *r* = 0.15, *P *= 0.028; Fig. 2G). These data are suggestive of a causal relationship between *NOVA1* and ER^+^ BC-specific ASEs, although further experimental studies are needed to confirm this.

Vice versa, expression of the *serine/arginine-rich splicing factor 12* (*SRSF12)* was 12 times higher in TNBC versus ER^+^ BC (FDR = 5.3e-6; Fig. 2F and H). In TCGA, *SRSF12* also exhibited significantly increased expression in TNBC (Supplementary Fig. S2B and D). To investigate which genes are subject to AS by *SRSF12*, we correlated PSI values of each ASE across cancer cells with *SRSF12* expression in each individual cell. Overall, among 9020 ASEs with detected junction-reads in at least 200 cells, 785 correlated significantly with *SRSF12* expression (Spearman’s correlation test with Benjamini–Hochberg adjusted, FDR < 0.05). Interestingly, these ASEs were 3-fold enriched for BC subtype-specific ASEs (Fig. 2I). For most of these ASEs (98%), *SRSF12* expression correlated with the alternative isoforms preferred in TNBC, consistent with the upregulation of *SRSF12* in TNBC (binomial test, *P *< 2.2e-16) (Fig. 2J). Next, we also applied JAseC to bulk RNAseq data from TCGA (without considering cell barcodes or UMIs). This confirmed a 2.6-fold enrichment of *SRSF12*-correlated ASEs in the subtype-specific group, with 97% of correlated ASEs changing in the expected direction (Supplementary Fig. S2E and F). *SRSF12* is known to elicit exon 4 skipping of the *Cdc2*-like kinase 1 (*CLK1*) [64]. Interestingly, kinase activation of *CLK1* has been observed in various cancer types where it correlates with worse prognosis, but skipping of exon 4 prevents *CLK1* from being activated [65]. In our data, exon 4 skipping was higher in TNBC (PSI = 0.431) than ER^+^ BC (PSI = 0.0279; likelihood ratio test in binomial GLM, *P *= 0.0219). Overall, this demonstrates that studying AS at single-cell level can discover which splicing factors are implicated in the phenotypic diversity of a given cancer type. However, further studies are needed to disentangle causal relationships from observed correlations.

### ASEs specific for tumor-infiltrating T cells

**Figure 3.**
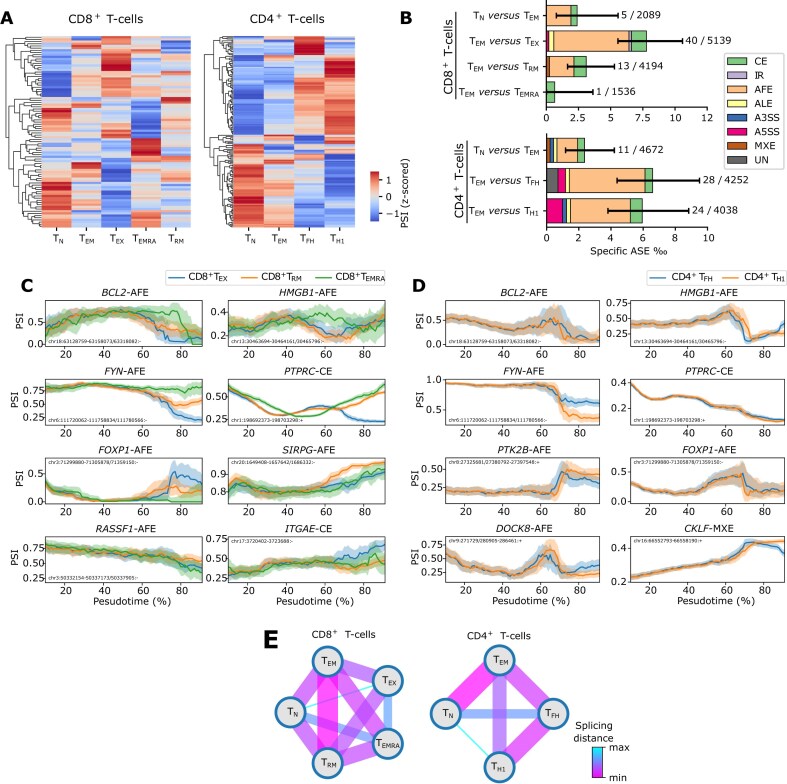
ASEs specific for T cells. (**A**) Heatmaps of specific ASEs differentially spliced among CD8^+^ (left panel) or CD4^+^ (right panel) T-cell subtypes. 140 or 224 differentially spliced ASEs between any two subgroups in CD8^+^ or CD4^+^ T cells, respectively, are shown. PSI values were logit transformed (0.01 offset) and *z*-score normalized in the heatmaps. (**B**) Proportion of specific ASEs amongst all detected ASEs in two subgroups of CD8^+^/CD4^+^ T cells, color coded by ASE type. Numbers of specific ASEs and detected ASEs are labeled beside each bar. Error bars indicate the 95% confidence intervals. (**C** and **D**) PSI trajectories of selected ASEs from genes chosen because they are known to be involved in immune signaling and regulation (*PTPRC, SIRPG, ITGAE, DOCK8, CKLF*, and *HMGB1*), or in modulating apoptosis and cell growth (*BCL2, FYN, RASSF1*, and *HMGB1*), across CD8^+^ (C) and CD4^+^ (D) T-cell subtypes, plotted along pseudo-time of development. Shaded ribbons indicate 95% confidence intervals, calculated using a sliding window (width = 20%). Gene name and AS type are labeled above each plot. (**E**) Graphs of splicing distance for CD8^+^ (left panel) and CD4^+^ (right panel) T-cell subtypes, based on Euclidean distances of logit-transformed PSI values from the specific ASEs among T-cell subtypes. Line thickness and color represent splicing similarity (thicker/warmer = more similar). T_N_: naïve T cell; T_EM_: effector-memory T cell; T_EX_: exhausted T cell; T_EMRA_: effector-memory re-expressing CD45RA T cell; T_RM_: tissue-resident memory T cell; T_FH_: follicular helper T cell; T_H1_: type-1 helper T cell.

We also explored ASEs that were specific for CD4^+^ or CD8^+^ T cells. Previous scRNAseq analyses identified various T-cell subtypes, including naïve (T_N_) cells, which differentiate toward activated effector-memory (T_EM_) cells and continue differentiating toward either programmed cell death protein-1 (PD1^+^)-expressing, exhausted (T_EX_) or effector-memory re-expressing CD45RA (T_EMRA_) T-cells [23]. Likewise, CD4^+^ T_EM_-cells differentiate toward either follicular helper T-cells (T_FH_) or type-1 T-helper cells (T_H1_). Out of the 5 702 ASEs detected in T-cells, 88 ASEs (in 84 genes) were specific to one of these CD8^+^ subtypes, while 161 (in 153 genes) were specific to one of the CD4^+^ subtypes (Fig. 3A, and Supplementary Tables S4 and S5). AFEs and CEs represented ~46% and 31% of these ASEs, respectively (Fig. 3B). Next, we assessed these ASEs along the differentiation trajectories of these CD8^+^ and CD4^+^ T cells (Fig. 3C and D) [23]. We explored splicing of the protein tyrosine phosphatase receptor type C (*PTPRC* or CD45), which functions as a well-known gatekeeper of T cells, whereby the long CD45RA isoform (exon 4 retained) suppresses immune responses and is typically expressed by naïve T-cells, while the short CD45RO isoform (exon 4 excluded) enables TCR signaling in response to high-affinity antigens and enhances cytokine production [66–68]. In our data, we could confirm that CD45 was differentially spliced along both CD4^+^ and CD8^+^ T-cell trajectories, whereby as expected CD45RA was prominent in T_N_ cells and CD45RO in T_EM_ and T_EX_ cells (Supplementary Fig. S3A). We also identified other genes with well-established functions in T cells exhibiting novel differentially spliced isoforms along these lineages, including the tumor-reactive T-cell marker *integrin subunit alpha E* (*ITGAE*) [69–71] and the proinflammatory cytokine *high mobility group box 1* (*HMGB1*) [72].

Analysis of splicing distances (i.e. Euclidean distances of logit-transformed PSI values) between T-cell subtypes recapitulated these trajectories. In CD8^+^ T cells, distances between terminal T_EX_- or T_EMRA_-cells were rather small, while distances between terminal (T_EX_- and T_EMRA_ cells) and naïve T_N_ cells were large, hence supporting that AS dynamically changes along these trajectories. In CD4^+^ T cells, similarly, T_N_ were close to T_EM_-cells but distant from terminally differentiated follicular helper T cells (T_FH_) or type 1 T-helper cells (T_H1_) (Fig. 3E). Finally, we observed that genes with ASEs specific for CD8^+^ T cells were enriched for biological processes involved in cell proliferation including “mitotic spindle” (*NUMA1, BIN1*, and *AKAP13*), “G2M checkpoint” (*CHEK1, SRSF2*, and *NUMA1*), as well as “cell aging” (*BCL2, MAP2K1*, and *ERCC1*) [73]. This is consistent with T cells undergoing proliferation and differentiation along the trajectories (Supplementary Fig. S3B and C).

### Validation of AS detection and quantification using stLFR and scATACseq

**Figure 4.**
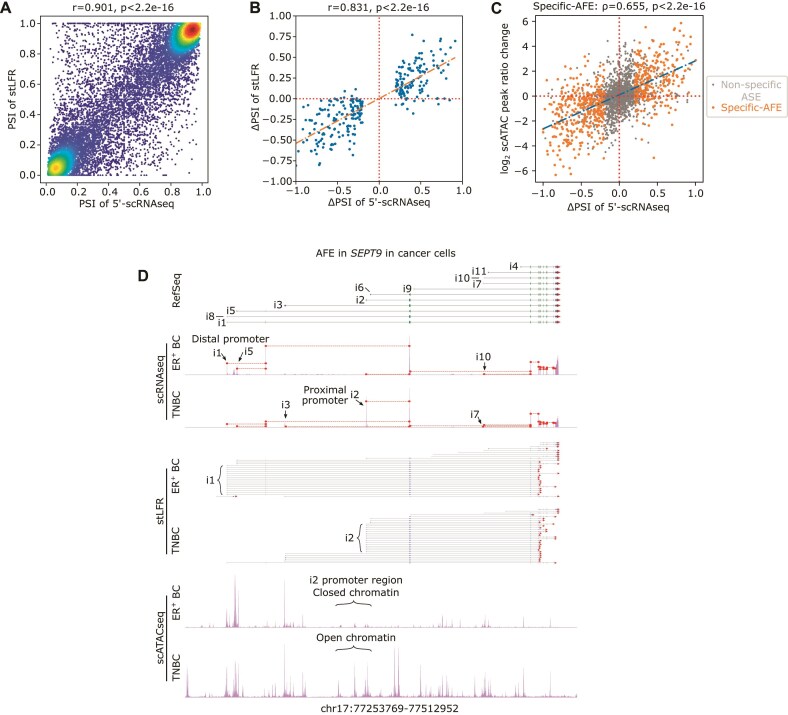
Validation of ASEs using parallel stLFR and scATACseq. (**A**) Scatter plot of PSI values from ASEs, showing the correlation between the 5′ scRNAseq data (*x*-axis) and the stLFR data (*y*-axis). Each dot indicates the PSI value of an ASE calculated by the combined reads of all four samples. A total of 15 343 ASEs were included, each with inclusion and exclusion read number ≥10 in both platforms, and with the PSI 95% confidence intervals inside the range of 0.01 to 0.99 in the 5′-scRNAseq data. (**B**) Scatter plot of the ΔPSI (cancer cells minus stromal cells), showing the correlation between the 5′-scRNAseq data (*x*-axis) and stLFR data (*y*-axis). A total of 198 ASEs with inclusion and exclusion read number ≥10 for both cancer cells and stromal cells of both platforms were used. (**C**) Scatter plot showing the correlation of ΔPSI of AFEs (*x*-axis) and change of Assay for Transposase-Accessible Chromatin (ATAC) peak ratio (*y*-axis) between cancer cells and stromal cells. The ATAC peak ratio was defined as the log2 fold changes of proximal peak to distal peak, each represented by CPM. Positive (negative) values on either axis indicate increased promoter usage or chromatin accessibility at proximal (distal) promoters in cancer cells. (**D**) Read coverage in *SEPT9* in the 5′ scRNAseq data (top panel) illustrating alternative promoter usage due to an AFE differentially spliced in ER^+^ BC versus TNBC in cancer cells from biopsies collected prior to treatment, drawn in the same style as Fig. 1H. *SEPT9* coverage in the stLFR data (middle panel) and chromatin accessibility differences in the scATACseq data (bottom panel) are also shown. In the scRNAseq panel, each *SEPT9* alternative isoform is labeled (“i1” – “i11”). In the stLFR panel, the top 30 longest reads in this region for each BC subtype are displayed. Each line represents a long read, with blue segments indicating aligned sequences, black segments indicating unaligned gaps, and red triangles at the 3′-end indicating the direction of the reads. The major isoform in each panel (“i1” or “i2”) is labeled in brackets. We failed to detect *circSEPT9*, as this isoform is not poly-adenylated and therefore cannot be detected in the 5′-scRNAseq protocol.

To validate ASEs identified in 5′-scRNAseq data, we applied in parallel stLFR technology [31] on full-length cDNA libraries of four BC samples. The stLFR technology tags sub-fragments of each PCR-duplicated full-length cDNA molecule with unique barcodes followed by massive-parallel sequencing. Based on stLFR and cell-specific UMI barcodes (10x Genomics), downstream bioinformatics analysis can merge short reads of the same transcript into a long read (see Supplementary Fig. S4 for distribution of merged read lengths). First, we assessed how many ASEs detected in the 5′-scRNAseq data were also supported by stLFR data, i.e. how many ASEs can be fully recovered by a long read for both the inclusion and exclusion isoform exon–exon boundary. This identified 78.4% of the known ASEs and 61.2% of the novel ASEs detected by 5′-scRNAseq in these four samples. For the high coverage ASEs (≥30 exclusion and ≥30 inclusion reads in the 5′-scRNAseq), 97.1% of the known and 86.1% of the novel ASEs were recapitulated in the stLFR data. ASEs that were missed had lower coverage in the stLFR data, suggesting they were missed due to low sequencing coverage. The observed degree of AS per major cell type and sample was highly correlated between both methods (*r* = 0.901, Fig. 4A). Likewise, ΔPSI values between cancer and stromal cells were strongly correlated (*r* = 0.831, Fig. 4B), confirming the validity of ASE detection and quantification with JAseC in 5′-scRNAseq.

Since AFEs were the second most frequent type of ASE and exhibited the highest cell type specificity, we validated them in more detail. Particularly, since AFEs are linked to alternative transcription start site (TSS) usage, we generated single-cell ATAC-sequencing (scATACseq) data on 12 tumor biopsies and explored TSS accessibility for all 2 775 detected AFEs (Supplementary Table S6). To comprehensively characterize the relationship between AFEs and chromatin accessibility, we determined the relative accessibility of proximal and distal promoters by computing the log2 ratio of the read counts of their corresponding scATACseq peaks (Materials and methods) and compared this to the respective AFE PSI value. Relative promoter accessibility and AFE PSI were strongly correlated when comparing cancer *versus* all stromal cells (Fig. 4C). Of the 2168 ASEs with detectable scATACseq peaks, 1445 (66.7%) exhibited concordant changes between relative accessibility and TSS usage. This concordance was 80.3% (497 out of 619) for ASEs specific for either cancer or stromal cells, hence confirming the validity of AFE detection with 5′-scRNAseq. We could also visually observe these differences in chromatin accessibility. For instance, the *septin 9* (*SEPT9*) isoform 2 (SEPT9_i2) was the preferred isoform, with regions of open chromatin surrounding a proximal promoter in TNBC (Fig. 4D). *SEPT9* is indeed known to promote BC progression in an isoform-specific manner, with different isoforms variably affecting cell migration, invasion, and metastasis via actin cytoskeleton and focal adhesion regulation [74–76].

### AS in the context of checkpoint immunotherapy

**Figure 5.**
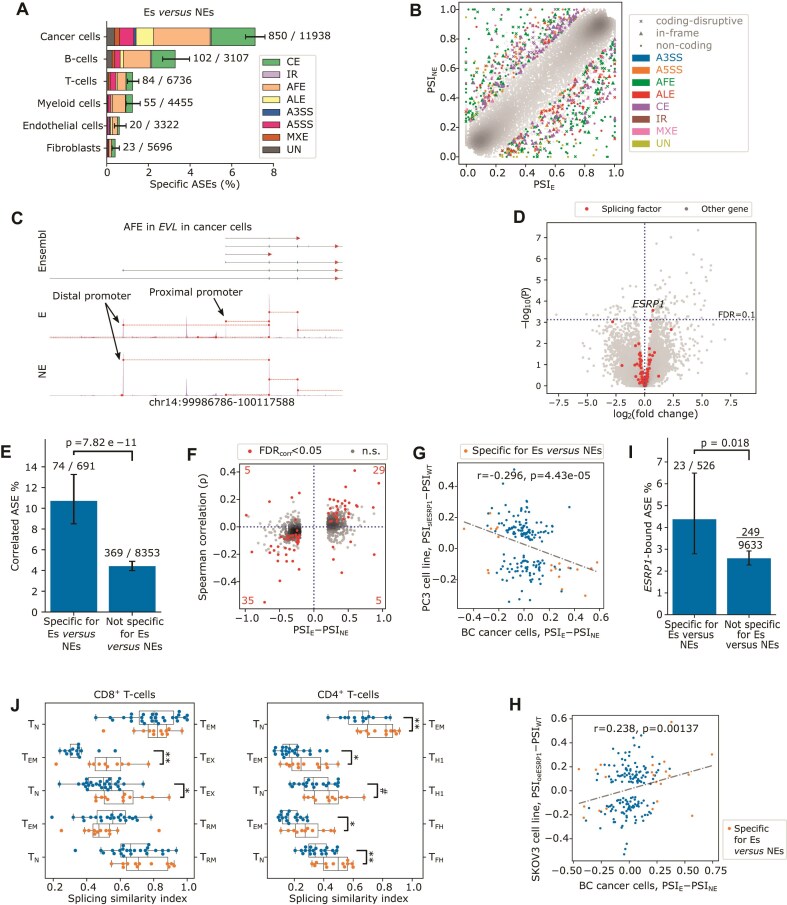
AS in the context of checkpoint immunotherapy. (**A**) Proportion of ASEs relative to all ASEs detected in pre-treatment samples comparing Es versus NEs, stratified by major cell type and color-coded by AS type. Numbers of specific ASEs versus all detected ASEs are labeled beside each bar. Error bars show 95% confidence intervals. (**B**) Scatter plot showing PSI values of detected ASEs in Es (*x*-axis) and NEs (*y*-axis). For expander-specific ASEs, colors indicate AS types, and marker shapes denote the predicted translational impact, as in Fig. 2D. (**C**) Read coverage of an AFE detected in *EVL* in cancer cells of pre-treatment Es versus NEs, depicted as in Fig. 1H. (**D**) Volcano plot showing differentially expressed genes comparing Es versus NEs in pre-treatment BC samples, tested using the DESeq2 likelihood ratio test with Benjamini–Hochberg adjustment (cutoff: FDR < 0.1) while controlling for BC subtype. Red dots highlight the 111 splicing regulators. (**E**) Proportion of ASEs with a significant Spearman’s correlation (FDR < 0.05) between PSI and *ESRP1* expression (CPM) at the single cell level across all cancer cells, stratified by whether the ASEs are differentiated between Es versus NEs in cancer cells. Fisher’s exact test *P*-values are shown; error bars represent 95% confidence intervals. (**F**) Scatter plot where each dot represents an ASE, showing the PSI value differences between Es versus NEs in cancer cells (*x*-axis), and the Spearman’s correlation (ρ) between its PSI and *ESRP1* expression at single-cell level among all cancer cells (*y*-axis). ASEs with significant correlations (Spearman’s correlation tests followed by Benjamini–Hochberg correction, FDR < 0.05) are highlighted in red, and their count in each quadrant is labeled in red number. (**G**) Scatter plot showing the ΔPSI of specific ASEs comparing Es versus NEs in cancer cells of BC samples (*x*-axis), and the ΔPSI of *ESRP1* siRNA knockdown versus control in the PC3 cell line (*y*-axis). (**H**) Scatter plot showing the ΔPSI of specific ASEs comparing Es versus NEs in cancer cells of BC samples (*x*-axis), and the ΔPSI of the SKOV3 *ESRP1* overexpression cell line versus control cell line (*y*-axis). (**I**) Proportion of *ESRP1*-bound ASEs (excluding AFEs) analyzed using public Cross-linking ImmunoPrecipitation or CLIP-seq data, stratified by whether ASEs are differentiated between Es and NEs in cancer cells. For both (G and H), the ρ and *P*-values of Spearman’s correlation were labeled. (**J**) Splicing similarity indexes in pre-treatment biopsies based on specific ASEs identified by comparing in CD8^+^ (left panel) or in CD4^+^ (right panel) T-cell subtypes. The compared T-cell subtypes are labeled on the left and right of each axis; T_N_: naïve T-cell; T_EM_: effector-memory T-cell; T_EX_: exhausted T-cell; T_RM_: tissue-resident memory T-cell; T_FH_: follicular helper T-cell; T_H1_: type-1 helper T-cell. CD8^+^ effector-memory re-expressing CD45RA T-cells (T_EMRA_) were excluded from this analysis since too few specific ASEs were identified due to the small number of single-cells belonging to this subcluster (*n* = 482, 2.39% of total CD8^+^ T cells). Each dot represents a sample. Similarity values closer to 0 (or 1) indicate that the AS profile of T cells in this sample more closely resembles the subtype labeled on the left (or right) of the axis, respectively. Significance: #: *P *< 0.1, *: *P *< 0.05, **: *P *< 0.01.

Given the emerging view that ASEs can contribute to tumor antigen load [10, 12, 77], we explored a role for cancer cell-specific ASEs during response to ICB. We compared patients with T-cell expansion following anti-PD1, identifying expanders or Es (considered treatment responders; *n* = 12) and patients without T-cell expansion (NEs, considered nonresponders: *n* = 28) [23]. We determined in each cell type ASEs specific for Es versus NEs in pre-treatment biopsies and found cancer cells to exhibit the largest number of specific ASEs (850 ASEs in 760 genes, or 7% of detected ASEs; Fisher’s exact test, *P *< 2.2e-16) (Fig. 5A and B, and Supplementary Table S7). Among these, AFEs were most frequent (*n* = 324, 38%), followed by CEs (*n* = 252, 30%), ALEs (*n* = 100, 12%), and A5SS (*n* = 81, 10%). For instance, we detected an AFE in *Enah/Vasp-like* (*EVL*), which in Es led to proximal promoter usage in ∼56% of transcripts, whereas in NEs the distal promoter was used in 97.2% (Fig. 5C). Notably, *EVL* is involved in actin polarization and plays a crucial role in suppressing invasion of ER^+^ BC by promoting the suppressive cortical actin bundles that inhibit membrane motility dynamics [78].

TNBC responds better to ICB than other BC subtypes [79]. Indeed, in our BC cohort, we included 18 TNBC and 19 ER^+^ BCs, of which 8 and 3 represented Es, respectively. Consequently, a considerable proportion of ASEs specific for Es versus NEs overlapped with those specific for TNBC versus ER^+^ BC (i.e. 60% for cancer cells, and 10%–26% for stromal cell types). To confirm that splicing differences were not merely due to BC subtype bias, we analyzed ASEs specific for Es versus NEs within TNBC and ER^+^ BC separately. Most of these showed consistent differences in the separate TNBC and ER^+^ BC analyses (ΔPSI_E-NE_ in ER^+^ BC and TNBC correlating more significantly for specific ASEs versus all ASEs detected; Supplementary Fig. S5A). This supports the notion that E versus NE splicing differences are not an artifact due to an imbalance in BC subtypes.

Differential gene expression analysis of splicing factors comparing Es versus NEs while controlling for BC subtype identified upregulation of the Epithelial Splicing Regulatory Protein-1 (*ESRP1*), which acts as a critical splicing regulator during epithelial–mesenchymal transition (Fig. 5D) [80]. When correlating PSI values of each ASE with expression of *ESRP1* in cancer cells, we found that 74 ASEs correlated significantly (FDR < 0.05). These were 2.5-fold enriched in the 691 ASEs specific for Es versus NEs and having junction-reads detected in >200 cells (Fig. 5E). For most of these 74 ASEs (86%), *ESRP1* expression correlated with the alternative isoforms preferred in Es, consistent with the upregulation of *ESRP1* in Es (binomial test, *P *< 2.2e-16; Fig. 5F). Next, we used public data in which siRNA-mediated *ESRP1* knockdown (PC3 cell line) or overexpression (SKOV3 cell line) was compared to wild-type conditions using bulk RNAseq. This revealed that ΔPSI values of ASEs significant in the E versus NE comparison showed a negative correlation with those obtained during knockdown (Pearson correlation: *r* = −0.3, *P *= 4.4e-5; Fig. 5G) [81], but a positive correlation when overexpressed (*r* = 0.24, *P *= 0.0014; Fig. 5H) [82]. We further validated these findings by performing *ESRP1* knockdown and overexpression in the MCF7 BC cell line, followed by bulk RNAseq. Similar correlations were observed (knockdown: *r* = −0.15, *P *= 0.034; overexpression: *r* = 0.22, *P *= 1.4e-4; Supplementary Fig. S5B and C). Finally, we used CLIP-seq data from a gastric cancer cell line [30], which showed that *ESRP1*-binding sites are enriched in ASEs specific for Es (odds ratio = 1.723, Fisher’s exact test, *P *= 0.018; Fig. 5I). Taken together, these observations support a role for *ESRP1* in determining AS differences between cancer cells from Es versus NEs.

Besides cancer cells, T cells also showed a high proportion of ASEs specific for Es versus NEs pre-treatment. Since we identified differentially spliced ASEs along CD4^+^ and CD8^+^ T-cell trajectories and since activation of T cells along these trajectories corresponds to T-cell expansion (E) [23], we also assessed whether T-cell specific ASEs could predict ICB response. Based on the 45 ASEs specific for effector-memory (T_EM_) versus exhausted (T_EX_) CD8^+^ T cells, we defined a splicing index for each tumor sample, assessing whether ASEs in CD8^+^ T cells were similar to T_EM_- (score = 0) or T_EX_-cells (score = 1). Both in pre- and on-treatment samples, this splicing index differed between Es versus NEs (Wilcoxon test, *P *< 0.01), with indices in Es being closer to CD8^+^ T_EX_-cells (Fig. 5J). Similarly, we identified 33 ASEs specific for CD4^+^ T_EM_- versus T_H1_/T_FH_-cells. When comparing Es versus NEs, we again observed that splicing indices differed (Wilcoxon test, *P *< 0.01; Figure 5J). Interestingly, both for CD8^+^ and CD4^+^ T cells, differences were more pronounced in on- than pre-treatment samples (Supplementary Fig. S5D). Overall, this confirms that ASEs detected in T cells from Es reflect the enrichment of CD8^+^ T_EX_- and CD4^+^ T_H1_/T_FH_ cells, which are well established to be tumor-reactive during ICB response [23].

### AS-derived tumor antigens predict response to ICB

**Figure 6.**
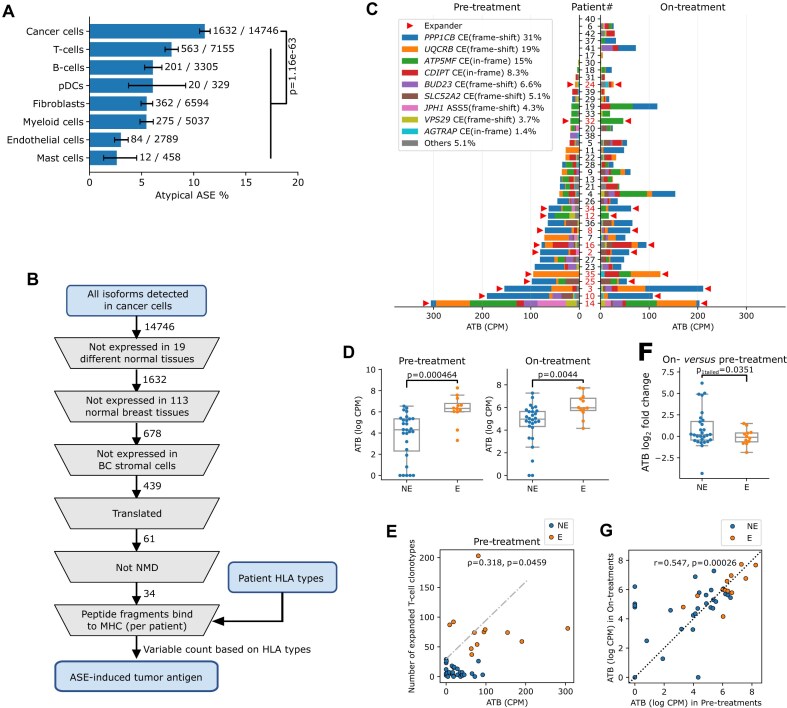
AS-derived tumor antigens predict response to ICB. (**A**) Proportion of atypical ASEs compared to all detected ASEs in the different cell types of BC samples. The number of tumor-specific atypical ASEs and detected ASEs are indicated on the right side. (**B**) Flowchart illustrating the pipeline to identify ASE-induced tumor antigens. The number of ASEs retained after each filtering step are indicated. (**C**) Bar plots showing the ATB for each BC pre-treatment sample. Each potential ASE-induced tumor antigen is represented by a different color. In the legend panel, the gene name, AS type, and percentage of samples in which the isoform was detected are indicated. In the middle column, sample IDs from Es and NEs are indicated in red and black, respectively. (**D**) ATB for each sample stratified by T-cell expansion. *P*-values depict the Wilcoxon test comparing Es versus NEs. (**E**) Scatter plot showing the correlation of ATB (*x*-axis) and the number of expanded T-cell clonotypes (*y*-axis). Red: Es; black: NEs. Spearman’s correlation coefficient (ρ) and significance (p) are indicated. (**F**) Log2 fold changes in ATB per sample during ICB therapy by comparing on- versus pre-treatment samples stratified for T-cell expansion. Positive values indicate an increase in ATB upon treatment. *P*-values from a one-tailed *t*-test are shown. Two patients for whom T-cell expansion data were not available were excluded. (**G**) Scatter plot showing the correlation of ATB in pre- (*x*-axis) versus on-treatment (*y*-axis) samples, colored by T-cell expansion. Pearson correlation coefficients (*r*) and their significance (*P*) are given.

Tumors can express isoforms that rarely occur in nonmalignant tissue and that therefore may be recognized by the host immune system as tumor antigens [9]. To identify such “atypical” isoforms, we compared ASEs detected in the paired biopsies of 42 BC patients to those identified in 19 normal adult tissues by bulk RNAseq (ENCODE RNAseq data; Supplementary Table S8) [83, 84]. For this, the following thresholds were applied: first, we selected ASEs detected in each cell type by scRNAseq (≥5 junction-reads) and only withheld those exhibiting an alternative isoform proportion >10-fold larger in cancer cells than the average across cell types. Then, we selected those ASEs exhibiting an alternative isoform proportion in cancer cells that was >4-fold larger than the maximal isoform proportion detected in normal tissue (see Materials and methods). We observed that cancer cells contained 1632 ASEs out of 14 746 ASEs detected by scRNAseq that fulfilled these conditions (Fig. 6A). Of these, we selected 439 ASEs that were also not detected in stromal cells by scRNAseq and not expressed in 113 normal breast tissues from TCGA. A parallel analysis, comparing only to normal bulk RNAseq tissue data, identified 917 atypical ASEs, demonstrating the value of filtering based on stromal cells from scRNAseq data as it excludes up to 50% of candidate atypical ASEs. Based on *in silico* translation, we predicted that 61 of these were translated, suggesting they potentially could encode for a tumor antigen (Fig. 6B and Supplementary Table S9). Among the 61 atypical ASEs, 27 of them were predicted to undergo NMD and therefore unlikely to represent tumor antigens. Of the remaining 34 ASEs, we could confirm by PCR that the normal and atypical isoforms of 22 ASEs were indeed expressed in tumor, but not healthy control tissue (Supplementary Fig. S6A).

Interestingly, CLIP-seq data showed that *ESRP1* (which was specifically upregulated in responders to ICB) can bind to 4 of the 34 atypical ASEs not undergoing NMD (i.e. in *ASAP3, BUD23, SLC34A3*, and *SYTL1*), which is significantly more than expected in case of random binding (odds ratio = 5.42; Fisher’s exact test, *P *= 0.0087), confirming that *ESRP1* could indeed contribute to the generation of tumor antigens. Three of the peptides generated by these ASEs (i.e. in *SLC52A2, JPH1*, and *HK3*) were also retrieved in publicly available label-free MS data from 125 TNBC tissues (ProteomeXchange ID:PXD000260; Materials and methods; Supplementary Fig. S6B) [85]. The unique peptide derived from the atypical ASE affecting *SLC52A2* was even detected in 22 proteomics samples.

Next, we investigated whether, similar to TMB, the abundance of the potential tumor antigen-inducing ASEs can predict response to ICB. For each tumor, we selected the 34 ASEs whose peptide fragments (8–11 mers) have a strong binding to the patient’s MHC as defined by the prediction netMHCpan tool [86, 87]. The “abundance” of each atypical ASE was subsequently estimated by multiplying its PSI with the expression level of the corresponding gene. The ATB was then obtained by summing abundances of all potential tumor antigen-inducing ASEs in each tumor (Fig. 6C). Interestingly, ATB was significantly higher in Es versus NEs for both pre-treatment (Wilcoxon test, *P *= 0.00046) and on-treatment (*P *= 0.0044) biopsies (Fig. 6D), while TMB did not differ [23]. When correcting for BC subtype, ATB was still increased in Es versus NEs (ANOVA with F-test, pre-treatment: *P *= 0.0029, on-treatment: *P *= 0.044). Pre-treatment, ATB was correlated with the number of expanded T-cell clonotypes (Spearman’s correlation, ρ = 0.32, *P *= 0.046; Fig. 6E). Compared to pre-treatment biopsies, ATB marginally decreased on-treatment in Es but not in NEs (Fig. 6F and G).

### ATB associates with immune activity in breast cancer TCGA

**Figure 7.**
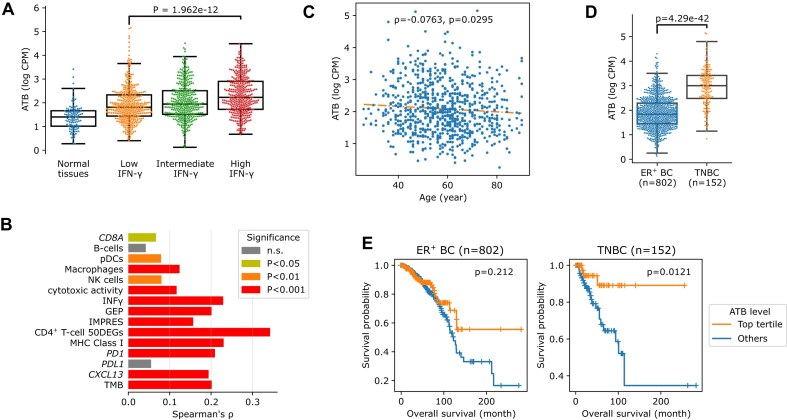
AS-derived tumor antigens in TCGA. (**A**) ASEs representing potential tumor antigens were considered to calculate the ATB in normal tissue, BC samples from TCGA stratified for the IFN-γ signature in the three indicated groups (low, intermediate, and high). (**B**) Spearman’s correlation between ATB and the indicated immune-related signature for TCGA BC tumors. The CD4^+^ T-cell 50 DEG signature was described by Bassez *et al.* [23]. GEP: T-cell inflamed gene expression; IMPRES: immuno-predictive score. (**C**) Scatter plot showing the negative correlation between ATB and patient age in TCGA BC tumors. The ρ and *P*-value of Spearman’s correlation tests are indicated. (**D**) ATB for each BC tumor from TCGA stratified for BC subtype. (**E**) OS curves for ER^+^ BC (left panel) and TNBC (right panel) in TCGA patients stratified by ATB (highest tertile versus others). *P*-values of log-rank tests comparing both groups are indicated.

To further characterize ATB in BC, we analyzed bulk RNAseq data of 1 099 primary breast tumors and 113 normal breast samples from TCGA and estimated ATB for each sample based on our 34 tumor antigen-inducing ASEs. Many atypical isoforms were detected in several tumors: 25 atypical isoforms were supported by ≥2 junction-reads in ≥50 tumors, while 14 isoforms exhibited proportions >5% in ≥50 breast tumors. Spearman’s correlation tests also showed that ATB associates with immune-reactive signatures, including interferon γ (IFN-γ) (ρ = 0.229, *P *= 21.44e-14), expression of MHC Class I genes (ρ = 0.23, *P *= 9.23e-15), T-cell inflamed gene expression (GEP) (ρ = 0.201, *P *= 1.59e-11), expression of *PD1* (ρ = 0.209, *P *= 2.09e-12), and *CXCL13* (ρ = 0.194, *P *= 8.07e-11) (Fig. 7A and B). Finally, these correlations were independent of TMB and tumor subtype (Supplementary Fig. S7A). Gene set enrichment analysis (GSEA) [29] on genes ranked according to their correlation with ATB revealed several enrichments, including with the immunity-related GO terms: immunoglobulin complex (*IGHG1, IGHG3*, and *CD79B*), T-cell receptor complex (*CD8B, CD3D*, and *TRBV28*), regulation of inflammatory response to antigenic stimuli (*PSMB4, IL20RB*, and *PSMA1*), as well as KEGG pathway allograft rejection (*GZMB, HLA-DOB*, and *HLA-F*) and antigen processing and presentation (*CALR, TAP2*, and *TAP1*). Additionally, RNA splicing related functions, such as KEGG pathway spliceosome (*SNRPG, ALYREF*, and *SNRPD1*) and GO component spliceosomal tri-snRNP complex (*SNRPG, SNRPD1*, and *FAM136A*) were strongly enriched (Supplementary Fig. S7B and Supplementary Table S11).

Further analyses showed that ATB in primary tumors was marginally negatively correlated with patient age (ρ = −0.0763, *P *= 0.0295) (Fig. 7C), while no differences were detected between tumor stages (Supplementary Fig. S7C). When stratifying tumors by BC subtype, we found TNBC to exhibit a higher ATB than ER^+^ BC (Fig. 7D). Furthermore, we observed that in TNBC, but not in ER^+^ BC, patients with a high ATB (i.e. top 33%) exhibited longer overall survival (OS; mean = 256 months) than other patients (mean = 143 months, log-rank test, *P *= 0.0121) (Fig. 7E), and this was independent of TMB (*P *= 0.038 for ATB when TMB is included as a covariate in the model). Overall, this suggests that ATB behaves similarly to TMB, whereby tumors with a high ATB burden are often immune-hot and associated with better patient outcome.

### AS-derived tumor antigens in advanced melanoma treated with anti-PD1

**Figure 8.**
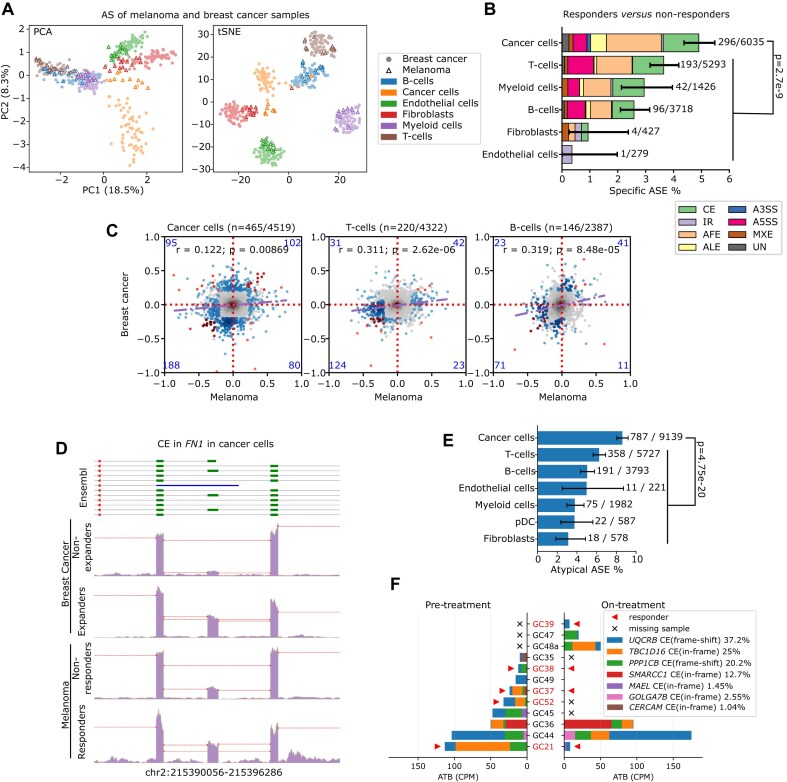
AS in a melanoma cohort treated with anti-PD1. (**A**) PCA (left) and tSNE (right) plots based on the PSI values of ASEs across major cell types in BC (light dots) and melanoma (hollow triangles). A total of 4066 ASEs with sufficient coverage (see Materials and methods) in all cell types and samples were considered. (**B**) Proportion of specific ASEs to all ASEs detected in pre-treatment melanoma comparing responders versus nonresponders, stratified by major cell type and color-coded by AS type. The numbers of response-specific ASEs versus all detected ASEs are labeled beside each bar. Error bar: 95% confidence interval. The Fisher’s exact test *P*-value indicates the difference in the proportion of specific ASEs between cancer cells and stromal cells. (**C**) Scatter plots showing the PSI changes between responders/Es and nonresponders/NEs in the pre-treatment samples of melanoma (*x*-axis) and BC (*y*-axis). Red, blue, and gray dots represent ASEs differentially spliced in both cancer types, in either cancer type, or in none of the cancer types, respectively. The labeled *r* and *P*-values represent Pearson correlation tests based on red and blue dots. (**D**) Read coverage of the CE in *FN1* in cancer cells of BC and melanoma, stratified by T-cell expansion or ICB response, depicted as in Fig. 1H. (**E**) Proportion of atypical splicing isoforms over all ASEs detected in pre-treatment melanoma stratified by major cell type. The numbers of atypical ASEs and detected ASEs are labeled beside each bar. (**F**) Bar plots showing ATB per melanoma sample, depicted as in Fig. 6F. In the middle column, sample IDs of each patient are highlighted in red and black, respectively.

We then explored whether ATB is associated with response to ICB also in other cancer types. We applied JAseC to 5′-scRNAseq data from advanced melanoma patients treated with anti-PD1. Particularly, we obtained data from 12 pre- and 12 on-treatment samples, 8 of which were paired (Supplementary Table S12) [53]. We integrated PSI values for each ASE in each cell type with those observed in the corresponding cell type in BC. Subsequently, PCA and tSNE plots revealed that ASEs clustered together regardless of cancer type, indicating shared cell type-specific splicing features between melanoma and BC (Fig. 8A). Most differences were observed in cancer cells, reflecting their different origin and genetic architecture. Indeed, in melanoma we identified 609 cancer cell-specific ASEs, 193 (31.7%) of which were also detected in BC, while for T-cell specific ASEs the overlap was larger (65.9%; Fisher’s exact test, *P *< 0.0001; Supplementary Fig. S8A).

Response according to RECIST was available 3 months after initiating anti-PD1 treatment in melanoma. When comparing ASEs between 5 responders (R) and 7 nonresponders (NR), we observed that cancer cells exhibited more ASEs specific for Rs versus NRs compared to stromal cell types (Fisher’s exact test, *P *= 2.7e-9; Fig. 8B). We then selected 6406 ASEs for which read coverage in pre-treatment tumors was sufficient to perform a comparative analysis between Rs versus NRs in melanoma, and Es versus NEs in BC. Among these ASEs specific in either dataset, ΔPSI values in T cells from melanoma correlated significantly with those observed in BC comparing Es versus NEs (Pearson correlation test for T cells: *r* = 0.31, *P *< 0.0001; Fig. 8C). For cancer cells, we observed a significant, but weaker correlation (*r* = 0.12, *P *= 0.009), which is expected as cancer cells in BC and melanoma have different origins and are patient specific. We identified 316 and 189 ASEs specific for Rs (or Es) versus NRs (or NEs) in melanoma or BC cancer cells, respectively, of which 28 were shared. For instance, the extracellular matrix protein *fibronectin* (*FN*1) contained an additional exon that was preferentially detected in Rs but skipped in NRs, both in melanoma and BC (Fig. 8D). *FN1* is known to play a role during tumor immune evasion by regulating extracellular matrix (ECM) remodeling [88, 89].Finally, when comparing ASEs detected in melanoma versus 19 normal tissues from ENCODE, we found that cancer cells contained 787 atypical ASEs (Fisher’s exact test, *P *= 4.748e-20) (Fig. 8E). A similar selection strategy as described for BC revealed 18 potential tumor antigen-inducing ASEs in melanoma. For two ASEs, we detected at least one atypical isoform in both BC and melanoma: one occurring in *UQCRB* and another in *the protein phosphatase 1 catalytic subunit beta* (*PPP1CB*) that was detected in up to 30 patients (Fig. 8F).

### The atypical *PPP1CB* isoform as a pan-cancer tumor antigen predicting response to ICB

**Figure 9.**
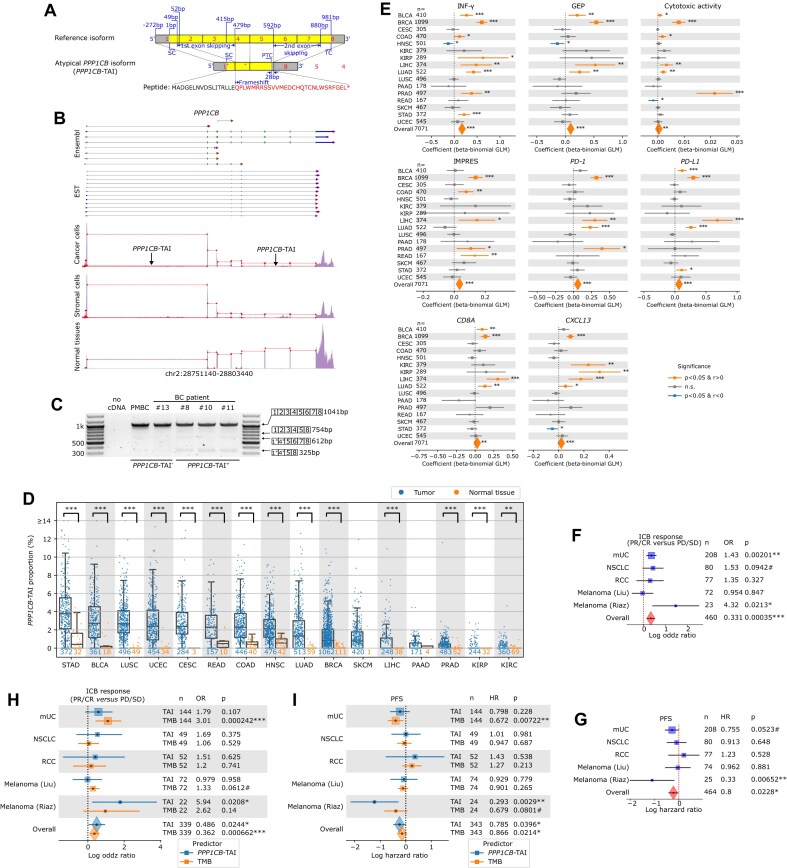
Characterization of the *PPP1CB* Tumor Antigenic Isoform (*PPP1CB*-TAI) at the pan-cancer level. (**A**) Schematic diagram for the splicing structure of the *protein phosphatase 1 catalytic subunit beta* (*PPP1CB*) tumor antigenic isoform or *PPP1CB*-TAI. SC: start codon; TC: termination codon; PTC: premature termination codon. Labeled nucleotide positions are numbered relative to the start codon (first nucleotide is indexed as 1). Asterisks denote truncated exons. (**B**) First panel: read coverage in the *PPP1CB* gene. Second panel: the near full-length EST showing that transcripts containing exon 5–8 read-junctions also contain exon 1*-4* read-junctions. Remaining panels: read coverage in BC cancer cells, stromal cells, and normal tissues plotted in the same style as Fig. 1H. (**C**) PCR results using primers designed on exon 1* and exon 8 to validate the PPP1CB isoforms. “no cDNA” refers to no-template control from patient #10. Based on the scRNAseq data patient sample #13 was predicted not to contain *PPP1CB*-TAI, while patient samples #8, #10, and #11 were predicted to contain the *PPP1CB*-TAI isoform. A linear gradient adjustment of brightness and contrast was applied along the *Y*-axis to correct for uneven fluorescence intensity using the ladder marker as reference. (**D**) Distribution of *PPP1CB*-TAI proportions in tumors from 16 solid cancer types and corresponding normal tissues from TCGA. Sample sizes for each group are indicated at the bottom. The significance labeled on the top depicts the splicing difference between tumors and normal tissues for each type of cancer estimated by the likelihood ratio test based on a beta-binomial GLM. STAD: Stomach adenocarcinoma; BLCA: Bladder Urothelial Carcinoma; LUSC: Lung squamous cell carcinoma; UCEC: Uterine Corpus Endometrial Carcinoma; CESC: Cervical squamous cell carcinoma and endocervical adenocarcinoma; READ: Rectum adenocarcinoma; COAD: Colon adenocarcinoma; HNSC: Head and Neck squamous cell carcinoma; LUAD: Lung adenocarcinoma; BRCA: Breast invasive carcinoma; SKCM: Skin Cutaneous Melanoma; LIHC: Liver hepatocellular carcinoma; PAAD: Pancreatic adenocarcinoma; PRAD: Prostate adenocarcinoma; KIRP: Kidney renal papillary cell carcinoma; KIRC: Kidney renal clear cell carcinoma. (**E**) Forest plots showing the association between *PPP1CB*-TAI proportion and each immune-related signature across individual cancer types and all types combined, using beta-binomial GLM. GEP: T-cell inflamed gene expression; IMPRES: immuno-predictive score. (**F**) Forest plot showing the association between *PPP1CB*-TAI proportion and ICB response in five cohorts involving anti-PD1/PDL1 treatment, using beta-binomial GLM. (**G**) Forest plot showing hazard ratios from Cox regression models for PFS associated with *PPP1CB*-TAI proportions. PR: partial response; CR: complete response; PD: progressive disease; SD: stable disease. (**H**) Forest plot showing the effects of *PPP1CB*-TAI proportion and TMB on ICB response across five cohorts involving anti-PD1/PDL1 treatment, using a multivariate GLM. (**I**) Forest plot showing the hazard ratios associated with *PPP1CB*-TAI proportion and TMB across five cohorts, using multivariate Cox regression models for PFS. In (E–I), the size of square markers represents the cohort sample size, and the horizontal lines indicate the 95% confidence intervals. Significance levels are visualized as follows: #: *P *< 0.1; *: *P *< 0.05; **: *P *< 0.01; ***: *P *< 0.001.

Because of its high frequency both in BC and melanoma, we further investigated the atypical ASE affecting *PPP1CB*. JAseC identified a junction between exon 5 and 8, but visual inspection of the read coverage revealed an additional noncanonical ASE upstream, which involved skipping a part of exon 1, both exons 2 and 3, as well as part of exon 4 (Fig. 9A and B). We describe this ASE as “exon 1*-4* junction.” PCR amplification of tumor cDNA confirmed that both ASEs are strongly linked (Fig. 9C), while investigation of the expressed sequence tag (EST) database in GenBank [90] confirmed that ESTs containing exon 5–8 junctions also contain exon 1*-4* junctions (Fig. 9B).

Next, we investigated the *PPP1CB* isoform that was predicted to be antigenic, *i.e*. the Tumor Antigenic Isoform in *PPP1CB* or *PPP1CB*-TAI, in bulk RNAseq data from 6 557 solid tumors in 16 cancer types, as well as 594 corresponding normal tissues. We observed that *PPP1CB*-TAI was present in each cancer type. Overall, 54.9% of tumors had an isoform proportion >1%, while 9.3% of tumors had an isoform proportion >5%. In the 13 cancer types with sufficient data on normal tissue available (≥5), isoform proportions were on average 9.24-fold higher in tumor versus normal tissue (beta-binomial test, *P *< 0.01; Fig. 9D). Similar results were observed when calculating *PPP1CB*-TAI abundance by multiplying gene expression (FPKM) with isoform proportion (Supplementary Fig. S9A).

We also determined in each of the 6557 tumors the correlation between the *PPP1CB*-TAI isoform proportion and the IFN-γ signature. Using beta-binomial GLM, we observed a significant positive correlation between *PPP1CB*-TAI and IFN-γ in 8 of 16 cancer types (likelihood ratio test, *P *< 0.05) (Fig. 9E). Other immune signatures, such as cytolytic activity, and expression of *PD1, PDL1, CXCL13*, and *CD8A*, also showed a positive correlation with the *PPP1CB*-TAI isoform proportion (Fig. 9E).

Finally, we investigated whether *PPP1CB*-TAI isoform proportions predict response to ICB based on public bulk RNAseq data from five clinical trials involving ICB. In total, 464 patients with mUC (trial IMvigor210) [91], NSCLC (trial POPLAR) [92], RCC (trial IMmotion150) [93], and advanced melanoma [94, 95] were used. While taking advantage of the paired-end reads available in these datasets, we first validated that the *PPP1CB*-TAI and exon 1*-4* isoforms were detected. In total, 11 501 read pairs were identified, of which 92.8% was derived from the normal isoform, 6.31% from the isoform containing skipping of “exon 6–7” as well as “exon 1*-4* junction,” while reads containing either of the two isoforms accounted for only 0.607% or 0.247%, respectively. *PPP1CB*-TAI isoform proportions were significantly correlated with objective response rates in mUC and one melanoma cohort (Riaz *et al.*, 2017) (likelihood ratio test, *P *< 0.05), while in NSCLC the association was almost significant (*P *< 0.1). Across all five trials, *PPP1CB*-TAI isoform proportions also correlated with response (likelihood ratio test, *P *= 0.00035) (Fig. 9F and Supplementary Fig. S9B), while high *PPP1CB*-TAI isoform proportions were associated with improved PFS (HR = 0.8, *P *= 0.0228 across all five cohorts) (Fig. 9G and Supplementary Fig. S9C). These results support the potential role of *PPP1CB*-TAI as a pan-cancer marker predicting response to ICB. Additionally, to investigate whether the predictive power of *PPP1CB*-TAI and TMB are overlapping, we repeated these regression analyses on 343 patients for whom whole-exome-sequencing data were also available, allowing TMB to be assessed as a covariate. In a model combining *PPP1CB*-TAI and TMB, both covariates independently predicted ICB response (Fig. 9H; p_TAI _= 0.0244, p_TMB _= 0.0007) and PFS (Fig. 9I; p_TAI _= 0.0396, p_TMB _= 0.0214). Overall, this suggests that by simultaneously considering *PPP1CB*-TAI and TMB, we could enhance the predictive accuracy for ICB therapy.

## Discussion

Here, we developed a computational pipeline, JAseC, to explore AS in single-cell data generated by the 10x Genomics platform. We confirm that AS can be better assessed using 5′ compared to 3′-scRNAseq data. Although both library types yield biased read coverage distributions, respectively, at the 5′ or 3′ ends of genes, 5′-scRNAseq generates more reads mapping along the entire gene length. We presume the main source of the reads in the middle and 3′ region of genes arises due to template switch oligo (TSO) strand invasion, as previously described [96–98]. Additionally, the first and internal exons have on average shorter exon lengths than the last exons [99], which further increases the likelihood of detecting ASEs in 5′-scRNAseq data. Overall, we identified 66 332 ASEs in 84 breast tumors, which could be classified into seven different types of ASEs, including AFEs. We could confirm the latter by performing scATACseq, while linked-read sequencing and transcript reconstruction (stLFR) on a subset of samples showed similar PSI values for the other types of ASEs, hence confirming the robustness of detecting ASEs using JAseC. Interestingly, our results show that it is meaningful to identify all seven types of ASEs, including AFEs, as they often revealed differences in the various biological comparisons that we investigated.

With the recent boom in publicly available single-cell data, which are mostly generated using 10x Genomics-based short-read sequencing approaches, JAseC has the potential to yield novel insights into the role of AS in health and disease. While we acknowledge that long-read single-cell sequencing technologies provide a more accurate means to identify ASEs, these are currently still poorly adopted for single-cell data due to their inherently high cost. We also found that none of the existing bioinformatics methods were suitable to analyze 10x-based single-cell data. Indeed, the vast majority of established single-cell AS methods are specifically designed for Smart-seq2 (or other long-read technologies) and are not compatible with droplet based single-cell technologies, as they often fail to account for the biased read coverage characteristic of droplet-based data (Supplementary Table S13). Moreover, a detailed characterization of each ASE, including explicit frequency observations of each alternative isoform to enable rigorous statistical modeling, is often lacking. Indeed, these requirements cannot be fulfilled by the few existing tools applicable to droplet-based data, including SpliZ [20], Marvel [18], and SCASL [100]. For instance, although SpliZ can be applied to droplet-based data, it uses a different strategy to detect ASEs and generates abstract scores on a gene level basis to avoid having to characterize ASEs affecting each gene in detail [20].

Importantly, in single-cell data ASEs cannot be studied in the context of individual cells because the number of reads supporting them is too low. They are, however, remarkably similar within a given cell (sub)type, whereas between cell (sub)types they show remarkable diversity. This allowed us to reliably explore AS within clusters of cells belonging to the same cell type (or cell phenotype in the case of T cells and B cells) and to generate an atlas of ASEs specific for cell types residing within the tumor microenvironment. For instance, we observed that cancer cells contained most cell type-specific ASEs compared to all other stromal cells. This could be due to somatic mutations affecting *cis*-regulatory elements [101] or due to expression changes or DNA mutations in transcription factors (e.g. AFEs) or splicing factors [102]. As an example, we found the splicing factor *NOVA1* to be upregulated in ER^+^ BC versus TNBC. We identified 184 ASEs comparing ER^+^ BC versus TNBC, with PSI values of these ASEs correlating significantly with *NOVA1* splicing factor expression in individual cells. In TCGA, *NOVA1* was validated as the most significant differentially expressed splicing factor in ER^+^ BC tumors, with ΔPSI values of ASEs specific for ER^+^ BC versus TNBC correlating between TCGA and single-cell data. Knockdown of *NOVA1* followed by bulk RNAseq confirmed that ΔPSI values in cancer cells from ER^+^ BC versus TNBC patients showed a significant positive correlation with those obtained from wild-type versus *NOVA1* knockdown conditions. We obtained similar data for *SRSF12*, which was upregulated in TNBC versus ER^+^ BC. Overall, these observations suggest that the molecular subtypes of BC are at least partly driven by AS and raise the question of how targeting *NOVA1* and *SRSF12* would phenotypically change ER^+^ BC or TNBC tumors.

We also investigated AS in T-cell subtypes and observed numerous ASEs specific for one of these cell types. These splice events followed the pseudotime trajectories previously defined for T cells using gene expression analyses. Moreover, the genes affected by these ASEs involved several genes with known immunoregulatory functions (*PTPRC, ITGAE, BCL2*, and *HMGB1*). Overall, this suggests that T-cell specific splicing events are involved in the development and differentiation of these cells, contributing to their activation and exhaustion in tumors. However, when studying AS in the context of checkpoint immunotherapy, both in BC and melanoma, we found that response specific ASEs were most frequent in cancer cells where they were, at least in part, regulated by the splicing factor *ESRP1*. Indeed, *ESRP1* was the most prominent splicing factor upregulated in Es, while at single-cell level PSI values of response specific ASEs correlated significantly with *ESRP1* expression. Interestingly, overexpression or knockdown of *ESRP1 in vitro* affected AS in the same way as observed in patients. An intriguing question is therefore to what extent *ESRP1* expression in tumors directly correlates with outcome to ICB and could perhaps be targeted to increase responsiveness to ICB.

Finally, we also showed how ASEs could possibly function as tumor antigens and how availability of single-cell data could contribute to their discovery. By identifying isoforms that were unique to cancer cells but absent from stromal cells and normal tissues, we identified several “atypical ASEs.” When predicting their MHC class I binding affinity, we detected 34 candidate tumor-antigenic ASEs in our BC cohort. Intriguingly, several of these were shared among patients, prompting us to quantify ATB in individual tumors. ATB was higher in tumors from Es versus NEs, while in TCGA ATB was strongly associated with immune-reactive signatures (IFN-γ, *CXCL13*, expression of MHC Class I genes and 50 response-associated genes in CD4^+^ T cells). When stratifying by BC subtype, we found TNBC to exhibit a higher ATB than ER^+^ tumors. In TNBC, but not ER^+^ BC, patients with a high ATB exhibited improved survival. Overall, this suggests that ATB behaves similarly to TMB, whereby high ATB or TMB levels positively correlate with immunoreactivity and improve survival across cancer types. The top-abundant ASE-induced tumor antigen giving rise to an atypical isoform *PPP1CB*-TAI, contributed to 74% of the ATB in BC. Interestingly, *PPP1CB*-TAI was also identified in single-cell data on melanoma and within TCGA bulk RNAseq data it was also more frequent in tumors across cancer types compared to matched normal tissue. When exploring public bulk RNAseq data from five clinical trials involving ICB, we could confirm that *PPP1CB*-TAI significantly predicted response independently of TMB, confirming its role as a potential tumor antigen. This is particularly interesting, as widespread occurrence of an ASE-induced tumor antigen in various cancer types could be attractive for targeted therapies such as cancer vaccines or TCR-based strategies.

In conclusion, by developing a novel workflow to detect AS from scRNAseq data, we investigated the cellular heterogeneity and dynamics of RNA splicing in tumors before and during checkpoint immunotherapy. We characterized splice events and their potential regulators within cancer and stromal cell types. Particularly, *ESRP1* was involved in response to ICB, while a cancer cell-specific ASE, i.e. *PPP1CB*-TAI, could serve as a potential tumor antigen predictive of response to ICB. Taken together, our work sheds light on the diversity and critical functions of AS in tumors, particularly during cancer immunotherapy response.

## Supplementary Material

gkaf1171_Supplemental_Files

## Data Availability

Raw sequencing reads of BC 5′-scRNAseq experiments are accessible from the European Genome-phenome Archive (EGA) (study no. EGAS00001004809, data accession no. EGAD00001006608). The source code of JAseC is available on Zenodo (DOI: 10.5281/zenodo.15848593) and also on GitHub: https://github.com/lambrechtslab/JAseC. All detected ASE structures, PSI values, and junction counts for each cell type across BC samples are deposited and freely accessible via Zenodo. The processed counts of scATACseq peaks and stLFR validated ASEs are also available on this webserver. Raw sequencing reads of bulk RNAseq for *NOVA1* knockdown in BT549 cells, as well as for *ESRP1* knockdown and overexpression in MCF7 cells, are deposited on Gene Expression Omnibus (ID: GSE305636). Raw sequencing reads of melanoma 5′-scRNAseq experiments were accessible from EGA (study no. EGAS00001006488, data accession no. EGAD00001009291). Raw sequencing reads of TCGA RNAseq data were downloaded from dbGaP (no. phs000178.v11.p8) after obtaining approval. For the raw bulk RNAseq reads and clinical data of ICB treated cohorts, data from mUC, NSCLC, and RCC were also downloaded from EGA (study no. EGAS00001004343, RNAseq data no. EGAD00001006631, clinical data no. EGAD00001006630) after obtaining approval, while data from melanoma (Liu *et al.*) [94] were downloaded from dbGaP (no. phs000452.v3.p1) and data from melanoma (Riaz *et al.*) [95] were downloaded from GEO (no. GSE91061).
