# Supplementary material for: Single-cell mapping of alternative splicing linked to checkpoint immunotherapy response
Source: Nucleic Acids Res. 2025 Nov 20;53(21):gkaf1171. doi: 10.1093/nar/gkaf1171 (PMC12631129; doi:10.1093/nar/gkaf1171)
Supplement: gkaf1171_Supplemental_Files [file gkaf1171_supplemental_files.zip › Attached file_ Supplementary-Figures.pdf]

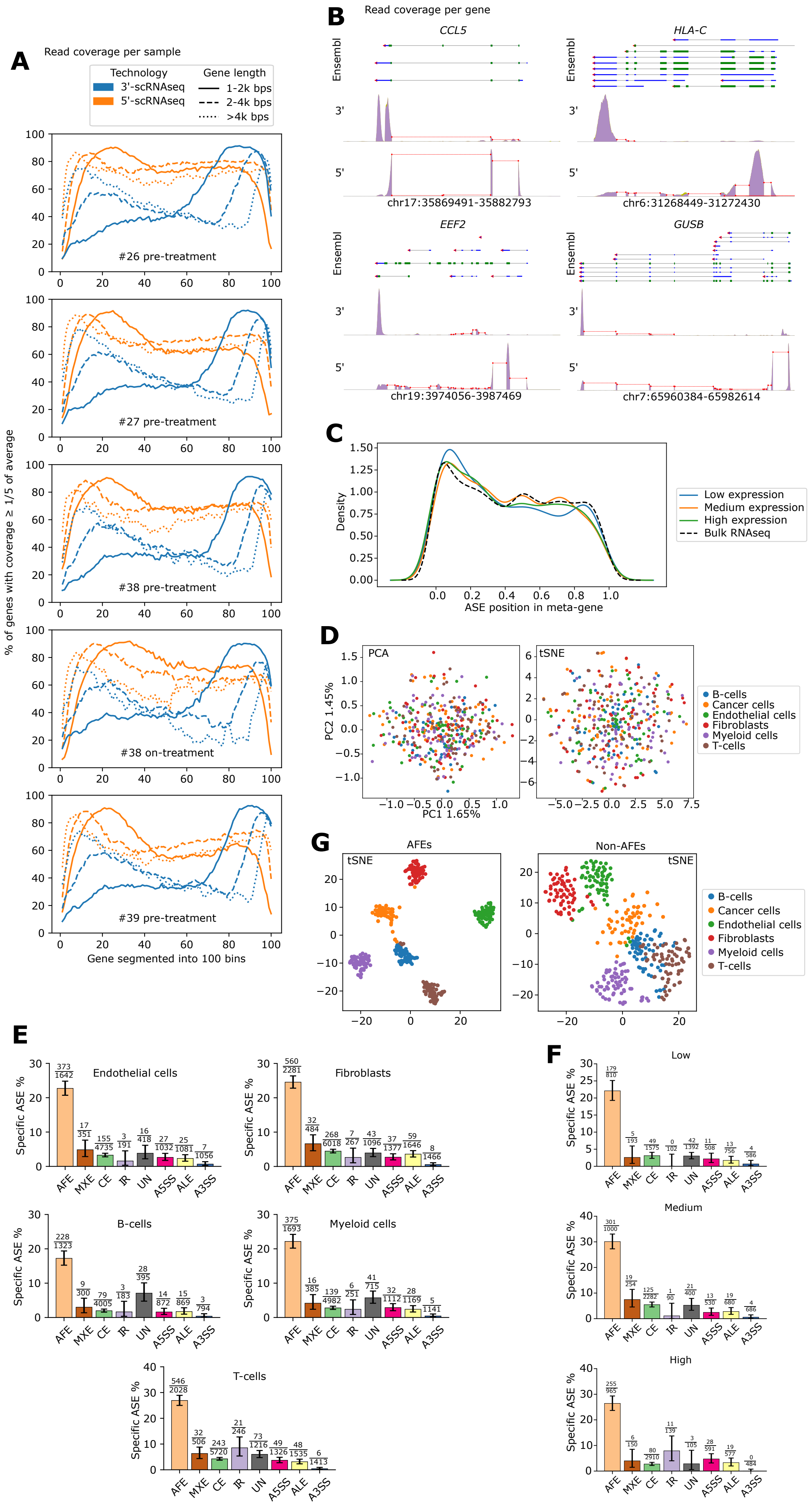
A
Read coverage per sample
Technology
3'-scRNAseq
5'-scRNAseq
Gene length
1-2k bps
2-4k bps
>4k bps
#26 pre-treatment
#27 pre-treatment
#38 pre-treatment
#38 on-treatment
#39 pre-treatment
% of genes with coverage ≥ 1/5 of average
Gene segmented into 100 bins
B
Read coverage per gene
CCL5
HLA-C
EEF2
GUSB
Ensembl
3'
5'
chr17:35869491-35882793
chr6:31268449-31272430
chr19:3974056-3987469
chr7:65960384-65982614
C
Density
ASE position in meta-gene
Low expression
Medium expression
High expression
Bulk RNAseq
D
PCA
tSNE
PC2 1.45%
PC1 1.65%
B-cells
Cancer cells
Endothelial cells
Fibroblasts
Myeloid cells
T-cells
G
AFEs
Non-AFEs
E
Endothelial cells
Fibroblasts
B-cells
Myeloid cells
T-cells
Specific ASE %
AFE
MXE
CE
IR
UN
A5SS
ALE
A3SS
F
Low
Medium
High

Supplementary Figure S1. A. Percentages of genes with good coverage in different gene regions, shown separately for each sample, depicted as in Figure 1A. **B.** Read coverage plots for *CCL5* (1,352 bp), *HLA-C* (1,542 bp), *EEF1* (3,158 bp), and *CD109* (9,031 bp), using parallel 3'-and 5'-scRNAseq data from five breast tumor samples. Annotation and coverage panels were depicted as in Figure 1H. **C.** Distribution of 3,740 cassette exons (CEs) along a normalized gene length in breast cancer (BC) 5'-scRNAseq as part of a meta-gene analysis stratified for low, medium, and high expression groups based on gene expression (CPM: counts per million). The black dashed line represents the distribution of 4,751 alternative splicing events (ASEs) along a normalized gene length identified in 1,221 BC bulk RNAseq data from TCGA. Only ASEs within a gene with length > 1k bps were considered, with their first and last intron junctions assigned as position 0 and 1, respectively. The area under the curve was normalized. **D.** Principal component analysis (PCA, left panel) and tSNE (right panel) plots based on randomized Percent-Spliced-In (PSI) values of ASEs from data aggregated by each cell type and BC sample. The same ASEs used as Figure 1G were included, but the inclusion and exclusion read numbers for each ASE were randomly shuffled. **E.** Proportion of ASEs specific to each cell type *versus* all other types, grouped by AS type, depicted as in Figure 1J. **F.** The proportion of cancer cell-specific ASEs stratified based on read coverage into three equal groups (low, medium, and high). ASE read coverage was defined by the sum of inclusion and exclusion reads used for calculating PSI. **G.** PCA and tSNE plots based on PSI values of ASEs from data aggregated by each cell type and BC sample, using only either 529 alternative first exons (AFEs) (left panel) or 3,718 non-AFE ASEs (right panel) with sufficient coverage across all cell type-sample combinations.

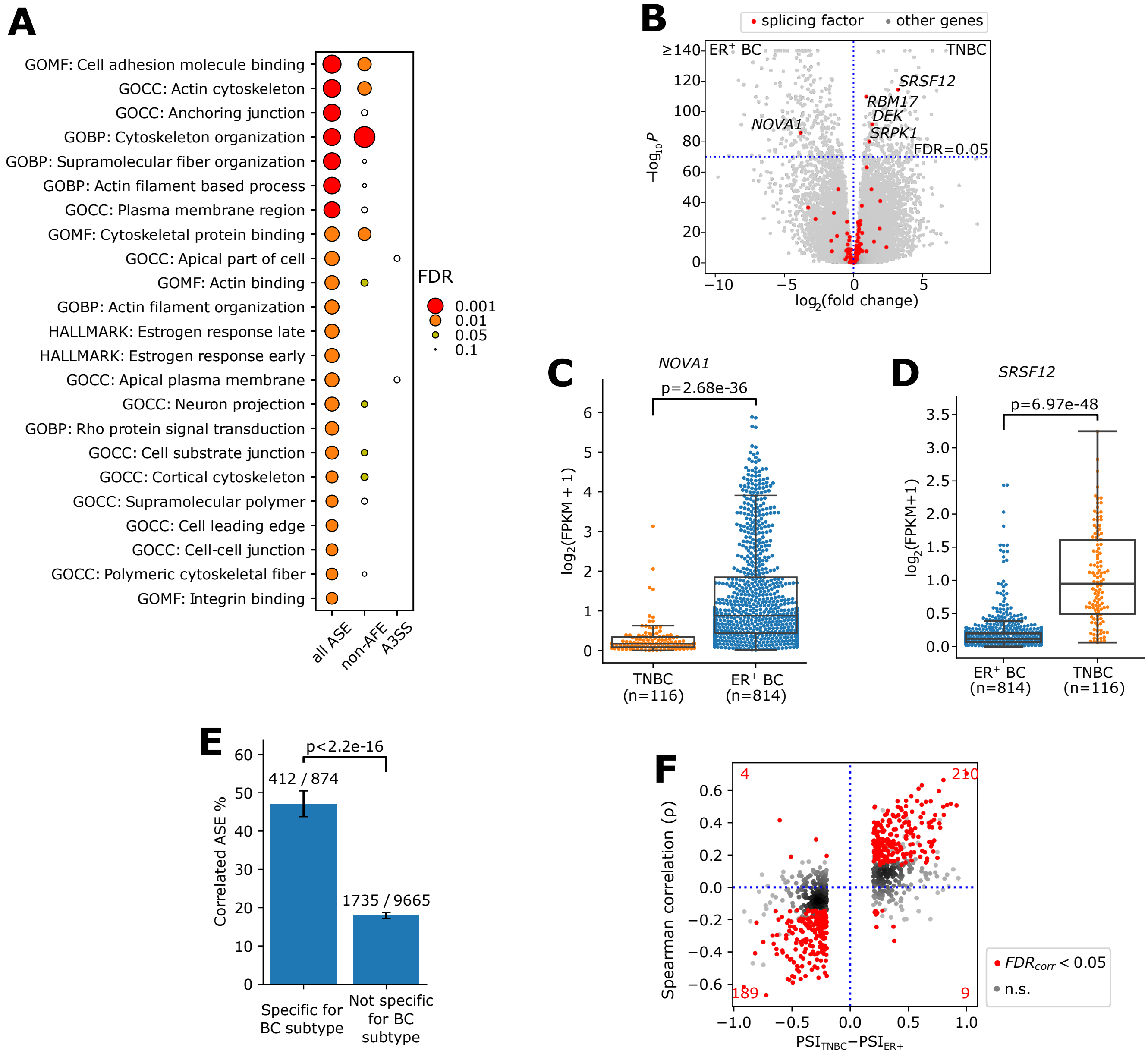

Supplementary Figure S2. A. Functional enrichment analysis of differentially spliced genes between ER^+ breast cancer (BC) and triple-negative BC (TNBC) in cancer cells. Dot size represents the significance level (only terms with FDR≤0.1 are shown). GOBP/MF/CC: gene ontology of biological process/molecular function/cellular component; KEGG: KEGG pathway database; HALLMARK: MSigDB hallmark gene sets. **B.** Volcano plot showing the differentially expressed genes between ER^+ BC and TNBC in the cancer genome atlas (TCGA) data. Red dots indicate the 111 splicing regulator genes. **C-D.** *NOVA1* (**C**) and *SRSF12* (**D**) gene expression levels in BCs from TCGA, stratified by BC subtype. Labeled p-values show the significances of Wilcoxon test. **E.** Proportion of ASEs with a significant Spearman correlation (FDR<0.05) between their Percent-Spliced-In (PSI) and *SRSF12* expression (CPM) values across 1,103 breast cancer (BC) samples from TCGA. Only ASEs with ≥10 junction reads in at least 50 samples, and with both alternative isoforms separately identified in at least 10 samples, are included. ASEs are stratified based on whether they are differentially spliced between estrogen receptor-positive (ER^+) BC and triple-negative BC (TNBC) in cancer cells. The Fisher's exact test p-value comparing between ASEs specific for BC subtype *versus* not specific for BC subtype is indicated. Error bars show 95% confidence interval. **F.** Scatter plot in which each ASE is represented by a dot. Differences between PSI values observed in TNBC *versus* ER^+ BC cancer (x-axis) are correlated with the Spearman's ρ for the correlation between PSI and *SRSF12* expression in BC from TCGA (y-axis). ASEs with a significant correlation

(FDR<0.05 according to a Spearman correlation test followed by Benjamini & Hochberg correction (FDR)) are shown as red dots. The number of red dots in each quadrant is labeled with a red number in the quadrant corner.

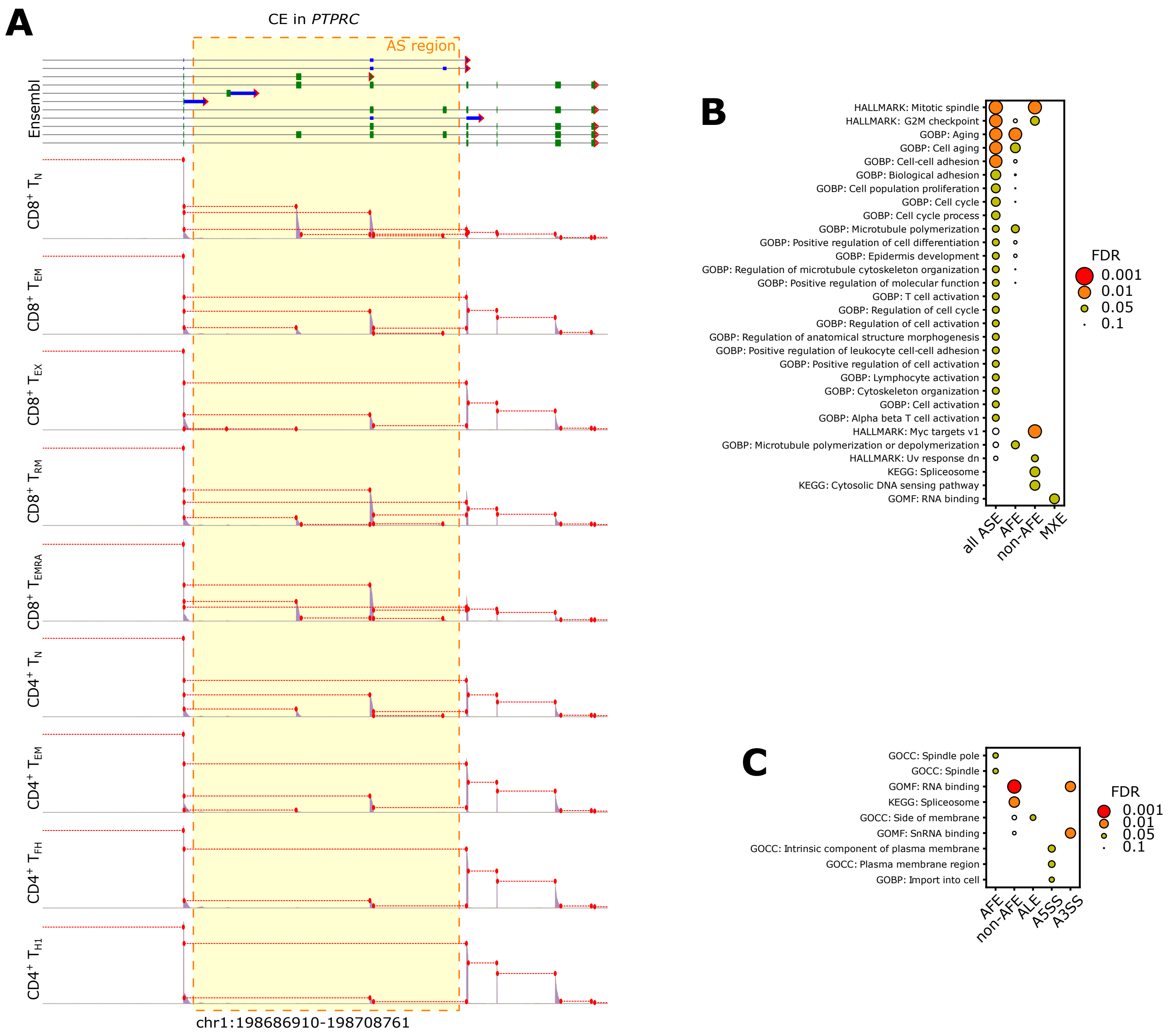

Supplementary Figure S3. A. Read coverage plots of specific alternative splicing events (ASEs) detected in *PTPRC* (or CD45) in different T-cell subtypes, depicted as in Figure 1H. **B-C.** Functional enrichment analysis of genes with specific ASEs among $CD8^{+}$ (**B**) or $CD4^{+}$ (**C**) T-cell subtypes, depicted as in Supplementary Figure S2A.

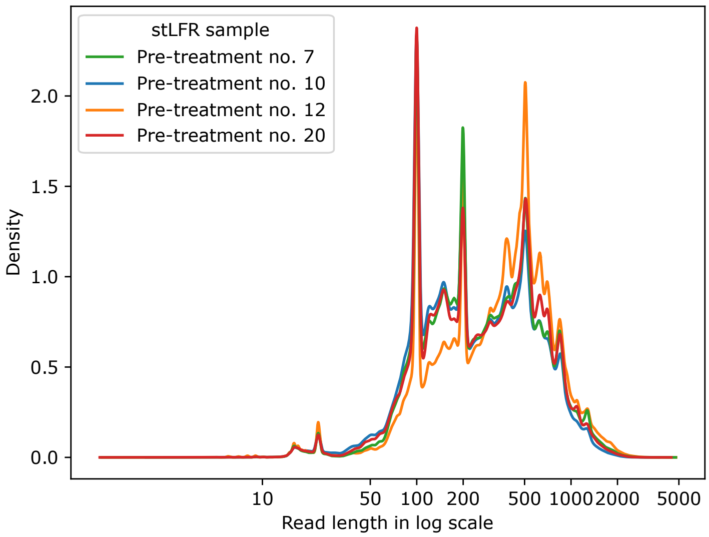

Supplementary Figure S4. Length distributions of UMI-linked single-tube long-fragment-read (stLFR) reads. For noncontinuous long reads, the uncovered gaps were not counted as read lengths. Sample #12 shows a longer average assembled read length due to its higher sequencing depth (sequenced 12 times) compared to the other three samples (each sequenced 4 times).

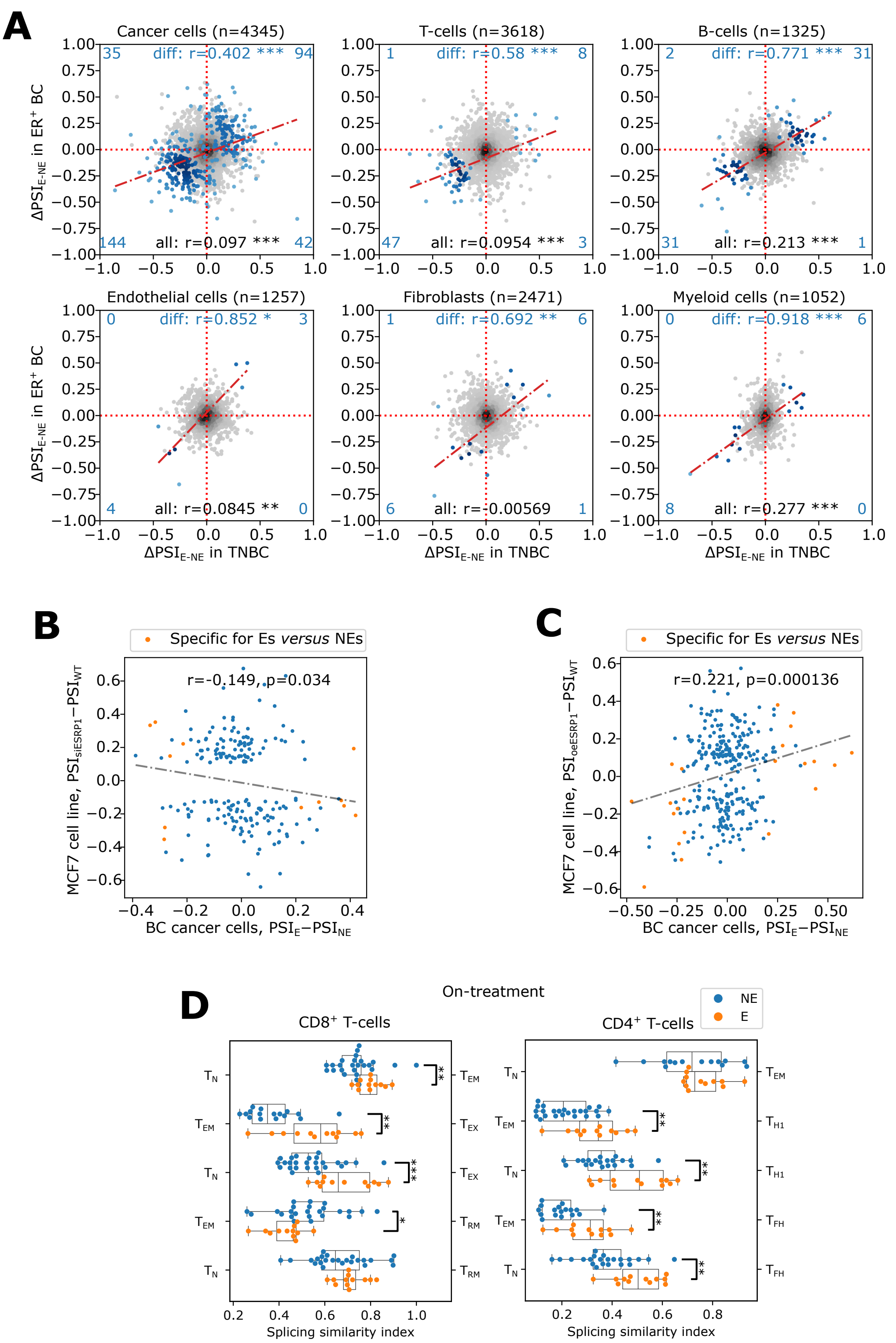

Supplementary Figure S5. A. Scatter plots showing the Percent-Spliced-In (PSI) differences between T-cell expanders (Es) and non-expanders (NEs) in triple-negative breast cancer (TNBC) samples (x-axis) *versus* in ER$^+$ breast cancer (BC) samples (y-axis). The alternative splicing events (ASEs) identified as Es-*versus*-NEs-specific in the combined pre-treatment samples are shown as blue dots. Pearson's correlation coefficients (r) for either blue dots (labeled as 'diff') or all dots (labeled as 'all') are labeled at the top or bottom of each panel. Significances: *: p<0.05; **: p<0.01; ***: p<0.001. The number of blue dots in each quadrant is labeled in the corners. Red-dash lines indicate the regression line for blue dots in each panel. **B-C.** Scatter plot showing the ΔPSI of response-specific ASEs comparing Es versus NEs in cancer cells of BC samples (x-axis), and the ΔPSI of *ESRP1* knockdown (**B**) or overexpression (**C**) *versus* control in the MCF7 cell line (y-axis). The coefficients and p values of Pearson correlation are labeled above. **D**. Splicing similarity indexes in on-treatment biopsies based

on specific ASEs identified by comparing in $CD8^+$ (left panel) or in $CD4^+$ (right panel) T-cell subtypes, plotted in the same style as Figure 5J.

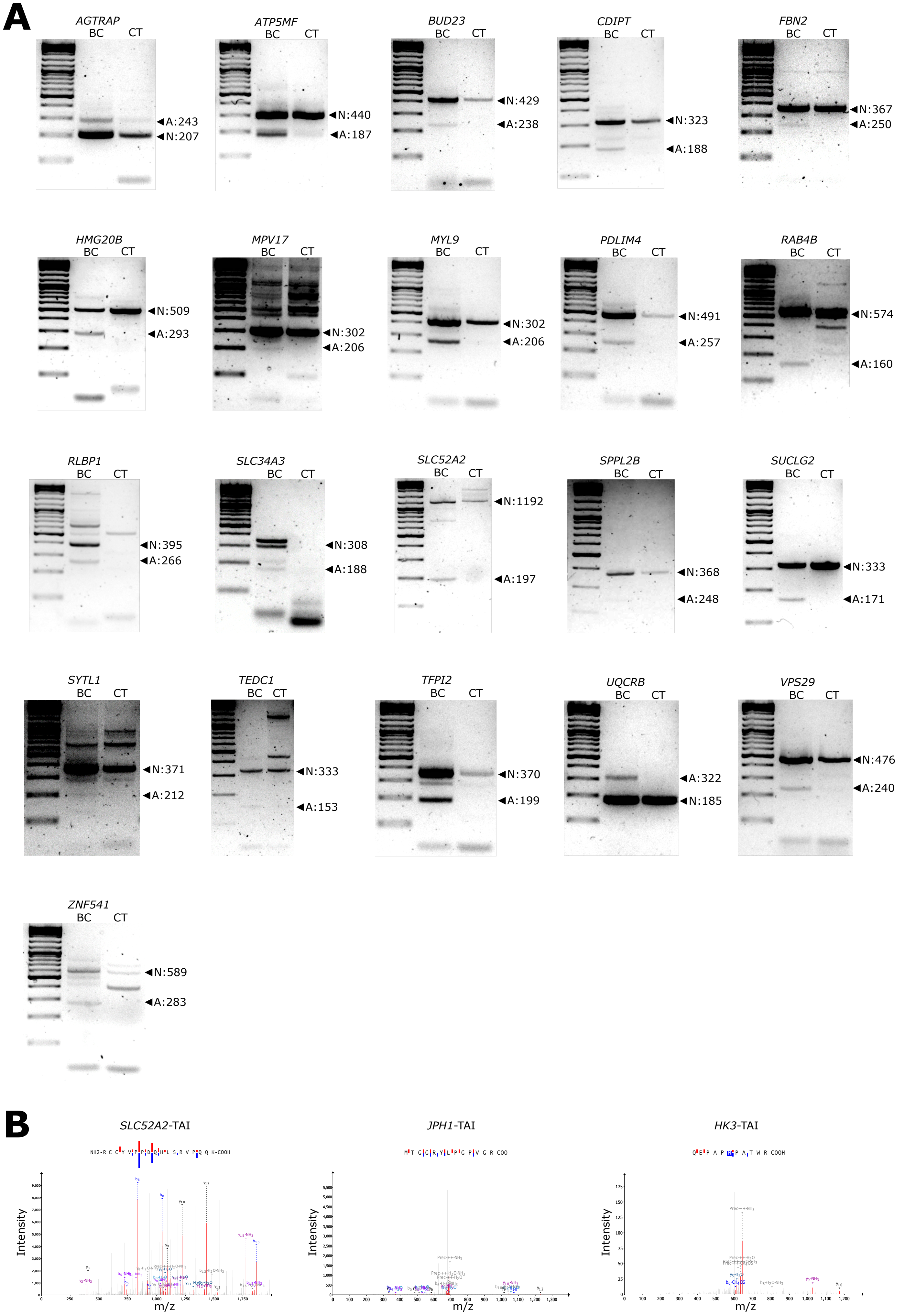
A
AGTRAP BC CT A:243 N:207
ATP5MF BC CT N:440 A:187
BUD23 BC CT N:429 A:238
CDIPT BC CT N:323 A:188
FBN2 BC CT N:367 A:250
HMG20B BC CT N:509 A:293
MPV17 BC CT N:302 A:206
MYL9 BC CT N:302 A:206
PDLIM4 BC CT N:491 A:257
RAB4B BC CT N:574 A:160
RLBP1 BC CT N:395 A:266
SLC34A3 BC CT N:308 A:188
SLC52A2 BC CT N:1192 A:197
SPPL2B BC CT N:368 A:248
SUCLG2 BC CT N:333 A:171
SYTL1 BC CT N:371 A:212
TEDC1 BC CT N:333 A:153
TFPI2 BC CT N:370 A:199
UQCRB BC CT A:322 N:185
VPS29 BC CT N:476 A:240
ZNF541 BC CT N:589 A:283
B
SLC52A2-TAI
JPH1-TAI
HK3-TAI
Intensity
m/z

Supplementary Figure S6. A. PCR validation of 22 atypical alternative splicing event (ASEs) identified in breast cancer (BC). On-treatment biopsies from patient #7 (*MPV17*, *RLBP1*, *SYTL1*, *TEDC1*), #11 (*PDLIM4*), #14 (*ATP5MF*, *BUD23*, *MYL9*, *PPP1CB*, *SPPL2B*, *UQCRB*, *VPS29*), #20 (*CDIPT*, *RAB4B*, *SLC34A3*, *SLC52A2*, *ZNF541*), and #31 (*AGTRAP*, *TFPI2*), as well as a pre-treatment biopsy from patient #29 (*HMG20B*, *FBN2*, *SUCLG2*) were used as a positive control (genes in brackets indicate that both isoforms of the ASE affecting this gene were detected in the scRNAseq data of this patient). For controls (CT), either peripheral blood mononuclear cells (PBMCs; for *AGTRAP*, *ATP5MF*, *HMG20B*, *MPV17*, *PPP1CB*, *RLBP1*, *SPPL2B*) or normal tissues (for the other ASEs) were used. Primers were designed on both sides of the ASEs to detect both the exclusion isoform (shorter product) and the inclusion isoform (longer product). The atypical and normal isoforms are labeled as 'A' and 'N', respectively. **B.** Representative mass spectrum plots, with mass-to-charge ratio on the x-axis and intensity on the y-axis, of the three peptide fragments specific to the BC atypical isoforms identified by screening public label-free proteomic mass spectrometry data from 125 TNBC tissues. TAI: tumor antigenic isoform.

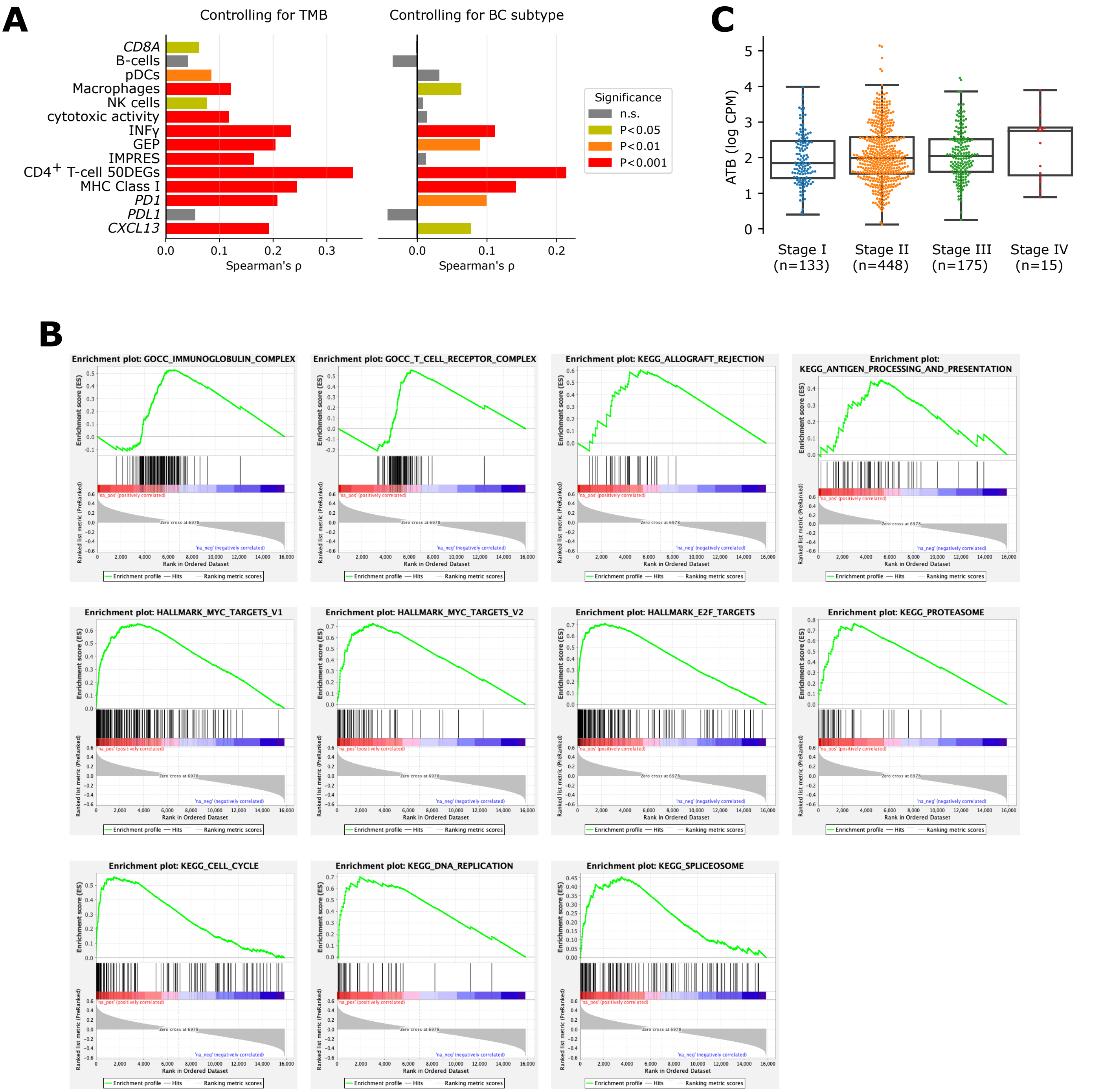

Supplementary Figure S7. A. Partial Spearman correlation between alternative splicing event (ASE)-derived tumor antigen burden (ATB) and each immune-related signature, controlling for the effect of tumor mutational burden (TMB) (left panel) or breast cancer (BC) subtype (right panel). IFNγ: interferon gamma; GEP: T-cell inflamed gene expression; IMPRES: immuno-predictive score. **B.** Gene set enrichment analysis (GSEA) enrichment plots for the genes correlated with ATB in the cancer genome atlas (TCGA) breast tumors. The 34 genes containing antigenic ASEs used for ATB calculation were excluded from the analysis. **C.** ATB distributions in TCGA primary breast tumors, stratified by tumor stages.

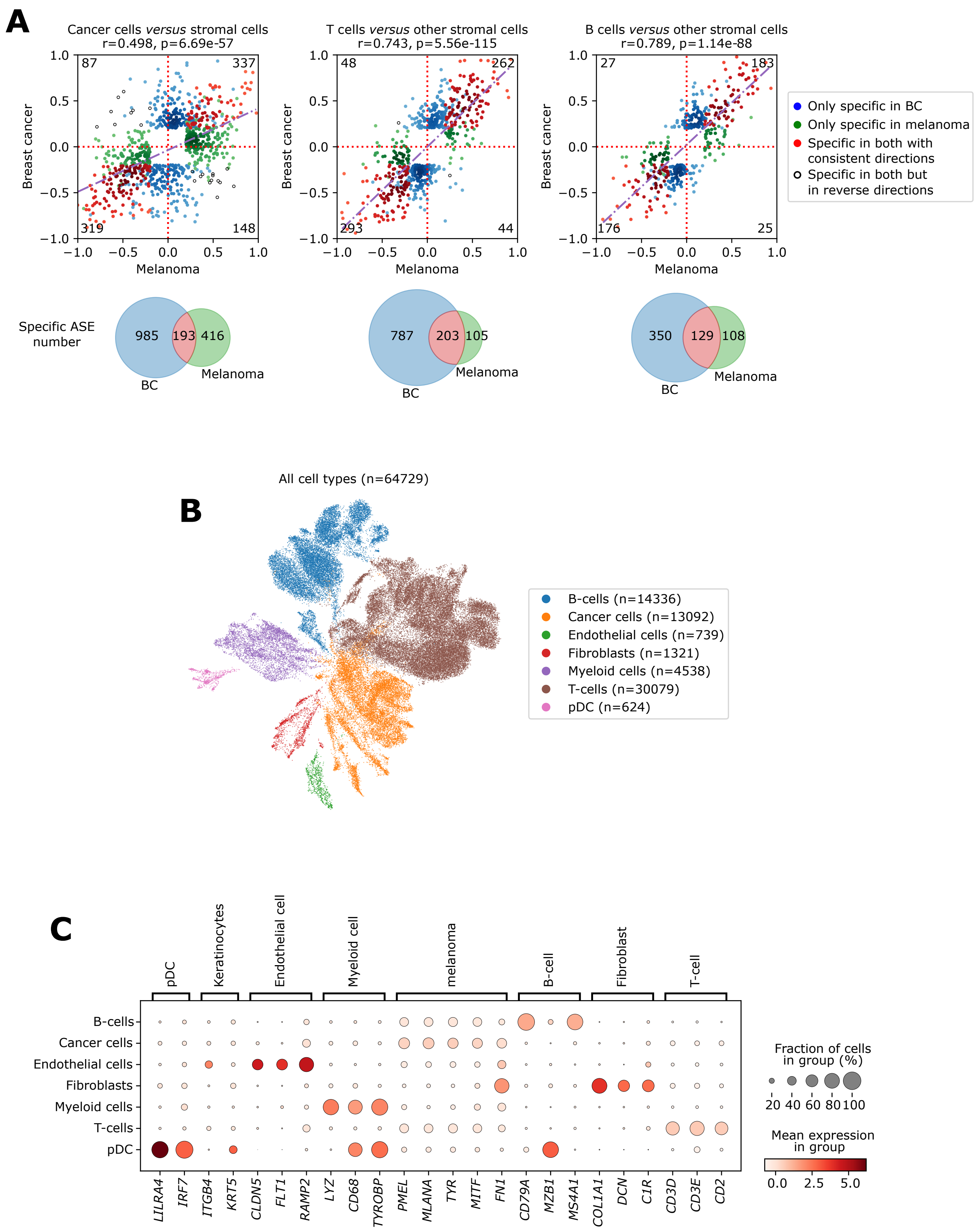

Supplementary Figure S8. A. Upper panels: scatter plots showing the Percent-Spliced-In (PSI) changes of specific alternative splicing events (ASEs) in cancer cells *versus* stromal cells (left panel), T-cells *versus* non-T-cell-stromal cells (middle panel), and B-cells *versus* non-B-cell-stromal cells (right panel) in the pre-treatments of melanoma (single-cell) (x-axis) and breast cancer (BC) (y-axis). The labeled r and p values represent the Pearson tests between two cancer types; lower panels: corresponding Venn diagrams showing the corresponding number of specific ASEs in either or both (with consistent change directions) cancer types. **B.**

UMAP plot of melanoma single cells based on gene expression profiles, colored by identified cell types. **C**. Dot plot showing the expression of selected marker genes across the identified cell types.

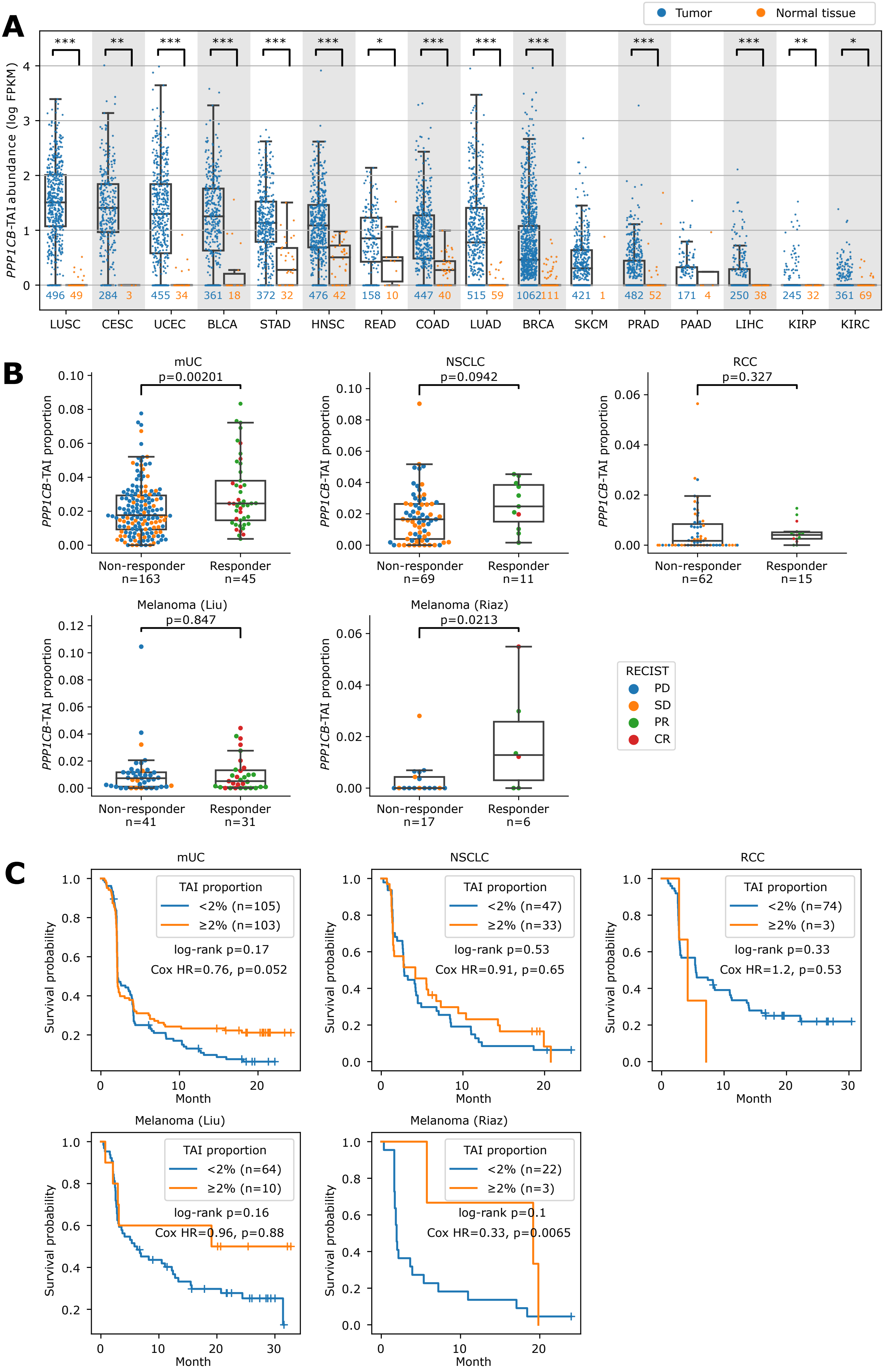

A
Tumor
Normal tissue
PPP1CB-TAI abundance (log FPKM)
LUSC CESC UCEC BLCA STAD HNSC READ COAD LUAD BRCA SKCM PRAD PAAD LIHC KIRP KIRC
496 49 284 3 455 34 361 18 372 32 476 42 158 10 447 40 515 59 1062111 421 1 482 52 171 4 250 38 245 32 361 69
*** ** *** *** *** *** * *** *** *** *** *** ** *
B
mUC p=0.00201
NSCLC p=0.0942
RCC p=0.327
Melanoma (Liu) p=0.847
Melanoma (Riaz) p=0.0213
PPP1CB-TAI proportion
Non-responder n=163
Responder n=45
Non-responder n=69
Responder n=11
Non-responder n=62
Responder n=15
Non-responder n=41
Responder n=31
Non-responder n=17
Responder n=6
RECIST
PD
SD
PR
CR
C
mUC
TAI proportion
<2% (n=105)
≥2% (n=103)
log-rank p=0.17
Cox HR=0.76, p=0.052
NSCLC
TAI proportion
<2% (n=47)
≥2% (n=33)
log-rank p=0.53
Cox HR=0.91, p=0.65
RCC
TAI proportion
<2% (n=74)
≥2% (n=3)
log-rank p=0.33
Cox HR=1.2, p=0.53
Melanoma (Liu)
TAI proportion
<2% (n=64)
≥2% (n=10)
log-rank p=0.16
Cox HR=0.96, p=0.88
Melanoma (Riaz)
TAI proportion
<2% (n=22)
≥2% (n=3)
log-rank p=0.1
Cox HR=0.33, p=0.0065
Survival probability
Month

Supplementary Figure S9. A. Distribution of *PPP1CB* tumor antigenic isoform (*PPP1CB*-TAI) abundance in tumors and corresponding normal tissues across 16 types of solid cancers from the cancer genome atlas (TCGA), depicted as in Figure 9E. TAI abundance was calculated as the product of *PPP1CB* gene expression and the '1-PSI' value of the *PPP1CB* ASE (to reflect abundance of its TAI isoform). STAD: Stomach adenocarcinoma; BLCA: Bladder Urothelial Carcinoma; LUSC: Lung squamous cell carcinoma; UCEC: Uterine Corpus Endometrial Carcinoma; CESC: Cervical squamous cell carcinoma and endocervical adenocarcinoma; READ: Rectum adenocarcinoma; COAD: Colon adenocarcinoma; HNSC: Head and Neck squamous cell carcinoma; LUAD: Lung adenocarcinoma; BRCA: Breast invasive carcinoma; SKCM: Skin Cutaneous Melanoma; LIHC: Liver hepatocellular carcinoma; PAAD: Pancreatic adenocarcinoma; PRAD: Prostate adenocarcinoma; KIRP: Kidney renal papillary cell carcinoma; KIRC: Kidney renal clear cell carcinoma. **B.** *PPP1CB*-TAI distributions in pre-treatment tumors from individual immune checkpoint blockade (ICB)-treated cohorts, stratified by response status. Between-group p-values were assessed using likelihood ratio test of beta-binomial generalized linear model (GLM). mUC: metastatic urothelial cancer; NSCLC: non-small cell lung cancer; RCC: renal cell carcinoma; PR: partial response; CR: complete response; PD: progressive disease; SD: stable disease. **C.** Progression-free survival (PFS) curves stratified by *PPP1CB*-TAI level in each cohort. Patients were grouped based on 2% cutoff of *PPP1CB*-TAI proportion in pre-treatment tumor biopsies.
