# Supplementary material for: Single-cell mapping of alternative splicing linked to checkpoint immunotherapy response
Source: Nucleic Acids Res. 2025 Nov 20;53(21):gkaf1171. doi: 10.1093/nar/gkaf1171 (PMC12631129; doi:10.1093/nar/gkaf1171)
Supplement: gkaf1171_Supplemental_Files [file gkaf1171_supplemental_files.zip › Supplementary_Table_S13.pdf]

Supplementary Table S13. Comparison of 10 methods to detect alternative splicing in single-cell data for various relevant parameters.

Method	Citation	Code	Input data	Unit detected	Used of junction reads	Used of non-junction reads	De novo ASE detection?	Supported ASE type	Outputs (not include downsteam analytics)
JAseC	n.a.	Julia	10x-based 5'-scRNAseq	ASE	Yes	Only IR (boundary reads)	Yes-Detecting ASE by junction reads (IR and MXE are by assembled annotation)	All types	PSI per cell group, inclusion & exclusion counts per cell
scASfind	(1)	R	SMART-seq2, VASA-seq	ASE (based on splicing node)	Yes (using Whippet)	No	No-Detection of ASEs annotated in Whippet index.	All types	PSI per cell pool
SCASL	(2)	Python	SMART-seq2, 10x-based 3'-scRNAseq	Alternative splice site, IR	Yes (using LeafCutter & FRASER)	Only IR	Yes-Detecting alternative 5'/3' splice site usage using FRASER	n.a.	Cell clustering
MARVEL	(3)	R	SMART-seq2, Fluidigm C1	ASE	Yes	Only IR	Yes/No-AFE & ALE: de novo detected, other ASE types: using predifined sets	All types	PSI, inclusion & exclusion counts per cell
MARVEL-AnnotateSJ.10x	(3)	R	10x-based 3'-scRNAseq	Junction	Yes	Yes (whole gene)	n.a.	n.a.	Ratio of junction count / gene count per cell
SpliZ	(4)	Python	10x-based 3'-scRNAseq, SMART-seq2	Gene	Yes	No	n.a.	n.a.	SpliZ score per cell
BRIE 1&2 (brie-count)	(5,6)	Python	SMART-seq2	ASE	Yes	Yes	No-Predefined based on gene annotation	CE	PSI, inclusion & exclusion counts per cell
SCATS	(7)	Python	SMART-seq2	Exon group	Unspecific	Yes	No-Base on gene annotation	n.a.	Counts per exon group per cell
VALERIE	(8)	R	SMART-seq2	ASE	Yes	Yes	No-Using predefined ASE sets	CE, MXE, IR, A5SS, A3SS	PSI, inclusion & exclusion counts per cell
Expedition	(9)	Python	SMART-seq2, Fluidigm C1	ASE	Yes	Yes	Yes-Detecting ASE by junction reads (using Outrigger)	CE, MXE	PSI, inclusion & exclusion counts per cell

Acronyms: ASE—alternative splicing event; CE—cassette exon; MXE—mutually-exclusive exon; IR—intron retention; A5SS—alternative 5'-splice site; A3SS—alternative 3'-splice cite; AFE—alternative first exon; ALE—alternative last exon; PSI—percent spliced-in.

Reference

1. Song, Y., Parada, G., Lee, J.T.H. and Hemberg, M. (2024) Mining alternative splicing patterns in scRNA-seq data using scASfind. *Genome Biol*, **25**, 197.
2. Xiang, X., He, Y., Zhang, Z. and Yang, X. (2024) Interrogations of single-cell RNA splicing landscapes with SCASL define new cell identities with physiological relevance. *Nat Commun*, **15**, 2164.
3. Wen, W.X., Mead, A.J. and Thongjuea, S. (2023) MARVEL: an integrated alternative splicing analysis platform for single-cell RNA sequencing data. *Nucleic Acids Res*, **51**, e29.
4. Olivieri, J.E., Dehghannasiri, R. and Salzman, J. (2022) The SpliZ generalizes 'percent spliced in' to reveal regulated splicing at single-cell resolution. *Nature Methods*, **19**, 307-310.
5. Huang, Y. and Sanguinetti, G. (2017) BRIE: transcriptome-wide splicing quantification in single cells. *Genome Biol*, **18**, 123.
6. Huang, Y. and Sanguinetti, G. (2021) BRIE2: computational identification of splicing phenotypes from single-cell transcriptomic experiments. *Genome Biol*, **22**, 251.
7. Hu, Y., Wang, K. and Li, M. (2020) Detecting differential alternative splicing events in scRNA-seq with or without Unique Molecular Identifiers. *PLoS Comput Biol*, **16**, e1007925.
8. Wen, W.X., Mead, A.J. and Thongjuea, S. (2020) VALERIE: Visual-based inspection of alternative splicing events at single-cell resolution. *PLoS Comput Biol*, **16**, e1008195.
9. Song, Y., Botvinnik, O.B., Lovci, M.T., Kakaradov, B., Liu, P., Xu, J.L. and Yeo, G.W. (2017) Single-Cell Alternative Splicing Analysis with Expedition Reveals Splicing Dynamics during Neuron Differentiation. *Mol Cell*, **67**, 148-161 e145.
